# Supplementary material for: Discovery and application of insertion-deletion (INDEL) polymorphisms for QTL mapping of early life-history traits in Atlantic salmon
Source: BMC Genomics. 2010 Mar 8;11:156. doi: 10.1186/1471-2164-11-156 (PMC2838853; doi:10.1186/1471-2164-11-156)
Supplement: Additional file 2 — Information on developed 76 locus single-run INDEL panel in Atlantic salmon. Information on fluorescence labeling, primer concentrations, PCR pooling and links to alignments, INDEL motifs and GENESCAN (Burge and Karlin 1997) predictions of genes/exons are available in html format. [file 1471-2164-11-156-S2.ZIP › Additionalfile2/table1329.html]

Contig Alignment

```
Base	A B C D E F G H I J K L M N O P Q R S T U V W X Y Z a b c d e f g h i j k l m n o   
1	. . . . . . . G . . . . . . . . . . . . . . . . . . . . . . . . . . . . . . . . .    
2	. . . . . . . G . . . . . . . . . . . . . . . . . . . . . . . . . . . . . . . . .    
3	. . . . . . . G . . . . . . . . . . . . . . . . . . . . . . . . . . . . . . . . .    
4	. . . . . . . G . . . . . . . . . . . . . . . . . . . . . . . . . . . . . . . . .    
5	. . . . . . . G . . . . . . . . . . . . . . . . . . . . . . . . . . . . . . . . .    
6	. . . . . . . A . . . . . . . . . . . . . . . . . . . . . . . . . . . . . . . . .    
7	. . . . . . . G . . . . . . . . . . . . . . . . . . . . . . . . . . . . . . . . .    
8	. . . . . . . C . . . . . . . . . . . . . . . . . . . . . . . . . . . . . . . . .    
9	. . . . . . . T . . . . . . . . . . . . . . . . . . . . . . . . . . . . . . . . .    
10	. . . . . . . G . . . . . . . . . . . . . . . . . . . . . . . . . . . . . . . . .    
11	. . . . . . . T . . . . . . . . . . . . . . . . . . . . . . . . . . . . . . . . .    
12	. . . . . . . A . . A . . . . . . . . . . . . . . . . . . . . . . . . . . . . . .    
13	. . . . . . . A . . A . . . . . . . . . . . . . . . . . . . . . . . . . . . . . .    
14	. . . . . . . G G . G . . . . . . . . . . . . . . . . . . . . . . . . . . . . . .    
15	. . . . . . . T T . T . . . . . . . . . . . . . . . . . . . . . . . . . . . . . .    
16	. . . . . . . C C . C . . . . . . . . . . . . . . . . . . . . . . . . . . . . . .    
17	. . G G G . . G G . G G . . . . . . . . . . . . . . . . . . . . . . . . . . . . .    
18	. . G G G . . G G . G G . . . . . . . . . . . . . . . . . . . . . . . . . . . . .    
19	. . A A A . . A A . A A . . . . . . . . . . . . . . . . . . . . . . . . . . . . .    
20	. . C C C . . C C . C C . . . . . . . . . . . . . . . . . . . . . . . . . . . . .    
21	. . A A A . . A A . A A . . . . . . . . . . . . . . . . . . . . . . . . . . . . .    
22	. . A A A . . A A . A A . . . . . . . . . . . . . . . . . . . . . . . . . . . . .    
23	. . G G G . . G G . G G . . . . . . . . . . . . . . . . . . . . . . . . . . . . .    
24	. . A A A . . A A . A A . . . . . . . . . . . . . . . . . . . . . . . . . . . . .    
25	. . T T T . . T T . T T . . . . . . . . . . . . . . . . . . . . . . . . . . . . .    
26	. . G G G . . G G . G G . . . . . . . . . . . . . . . . . . . . . . . . . . . . .    
27	. . G G G . . G G . G G . . . . . . . . . . . . . . . . . . . . . . . . . . . . .    
28	. . C C C . . C C . C C . . . . . . . . . . . . . . . . . . . . . . . . . . . . .    
29	. . G G G . . G G . G G . . . . . . . . . . . . . . . . . . . . . . . . . . . . .    
30	. . A A A . . A A . A A . . . . . . . . . . . . . . . . . . . . . . . . . . . . .    
31	. . C C C . . C C . C C . . . . . . . . . . . . . . . . . . . . . . . . . . . . .    
32	. . G G G . . G G . G G . . . . . . . . . . . . . . . . . . . . . . . . . . . . .    
33	. . A A A . . A A . A A . . . . . . . . . . . . . . . . . . . . . . . . . . . . .    
34	. . G G G . . G G . G G . . . . . . . . . . . . . . . . . . . . . . . . . . . . .    
35	. . C C C . . C C . C C . . . . . . . . . . . . . . . . . . . . . . . . . . . . .    
36	. . A A A . . A A . A A . . . . . . . . . . . . . . . . . . . . . . . . . . . . .    
37	. . A A A . . A A . A A . . . . . . . . . . . . . . . . . . . . . . . . . . . . .    
38	. . T T T . . T T . T T . . . . . . . . . . . . . . . . . . . . . . . . . . . . .    
39	. . G G G . . G G . G G . . . . . . . . . . . . . . . . . . . . . . . . . . . . .    
40	. . G G G . . G G . G G . . . . . . . . . . . . . . . . . . . . . . . . . . . . .    
41	. . T T T . . T T . T T . . . . . . . . . . . . . . . . . . . . . . . . . . . . .    
42	. . G G G . . G G . G G . . . . . . . . . . . . . . . . . . . . . . . . . . . . .    
43	. . G G G . . G G . G G . . . . . . . . . . . . . . . . . . . . . . . . . . . . .    
44	. . T T T . . T T T T T . . . . . . . . . . . . . . . . . . . . . . . . . . . . .    
45	. . G G G . . G G G G G . . . . . . . . . . . . . . . . . . . . . . . . . . . . .    
46	. . G G G . . G G G G G . . . . . . . . . . . . . . . . . . . . . . . . . . . . .    
47	. . A A A . . A A A A A A . . . . . . . . . . . . . . . . . . . . . . . . . . . .    
48	. . A A A . . A A A A A A . . . . . . . . . . . . . . . . . . . . . . . . . . . .    
49	. . T T T . . T T T T T T . . . . . . . . . . . . . . . . . . . . . . . . . . . .    
50	. . G G G . . G G G G G G . . . . . . . . . . . . . . . . . . . . . . . . . . . .    
51	. . G G G . . G G G G G G . . . . . . . . . . . . . . . . . . . . . . . . . . . .    
52	. . A A A . . A A A A A A . . . . . . . . . . . . . . . . . . . . . . . . . . . .    
53	. . A A A . . A A A A A A . . . . . . . . . . . . . . . . . . . . . . . . . . . .    
54	. . G G G . . G G G G G G . . . . . . . . . . . . . . . . . . . . . . . . . . . .    
55	. . T T T . . T T T T T T . . . . . . . . . . . . . . . . . . . . . . . . . . . .    
56	. . G G G . . G G G G G G . . . . . . . . . . . . . . . . . . . . . . . . . . . .    
57	. . G G G G . G G G G G G . . . . . . . . . . . . . . . . . . . . . . . . . . . .    
58	. . A A A A . A A A A A A . . . . . . . . . . . . . . . . . . . . . . . . . . . .    
59	. . T T T T . T T T T T T . . . . . . . . . . . . . . . . . . . . . . . . . . . .    
60	. . G G G G . G G G G G G . . . . . . . . . . . . . . . . . . . . . . . . . . . .    
61	. . G G G G . G G G G G G . . . . . . . . . . . . . . . . . . . . . . . . . . . .    
62	. . G G G G . G G G G G G . . . . . . . . . . . . . . . . . . . . . . . . . . . .    
63	. . G G G G . G G G G G G . . . . . . . . . . . . . . . . . . . . . . . . . . . .    
64	. . C C C C . C C C C C C . . . . . . . . . . . . . . . . . . . . . . . . . . . .    
65	. . A A A A . A A A A A A . . . . . . . . . . . . . . . . . . . . . . . . . . . .    
66	. . G G G G . G G G G G G . . . . . . . . . . . . . . . . . . . . . . . . . . . .    
67	. . C C C C . C C C C C C . . . . . . . . . . . . . . . . . . . . . . . . . . . .    
68	. . C C C C . C C C C C C . . . . . . . . . . . . . . . . . . . . . . . . . . . .    
69	. A A A A A . A A A A A A . . . . . . . . . . . . . . . . . . . . . . . . . . . .    
70	. G G G G G G G G G G G G . . . . . . . . . . . . . . . . . . . . . . . . . . . .    
71	. C C C C C C C C C C C C . . . . . . . . . . . . . . . . . . . . . . . . . . . .    
72	. C C C C C C C C C C C C . . . . . . . . . . . . . . . . . . . . . . . . . . . .    
73	. C C C C C C C C C C C C . . . . . . . . . . . . . . . . . . . . . . . . . . . .    
74	. C C C C C C C C C C C C . . . . . . . . . . . . . . . . . . . . . . . . . . . .    
75	. A A A A A A A A A A A A . . . . . . . . . . . . . . . . . . . . . . . . . . . .    
76	. G G G G G G G G G G G G . . . . . . . . . . . . . . . . . . . . . . . . . . . .    
77	. T T T T T T T T T T T T . . . . . . . . . . . . . . . . . . . . . . . . . . . .    
78	. G G G G G G G G G G G G . . . . . . . . . . . . . . . . . . . . . . . . . . . .    
79	. T T T T T T T T T T T T . . . . . . . . . . . . . . . . . . . . . . . . . . . .    
80	. T T T T T T T T T T T T . . . . . . . . . . . . . . . . . . . . . . . . . . . .    
81	. A A A A A A A A A A A A . . . . . . . . . . . . . . . . . . . . . . . . . . . .    
82	. T T T T T T T T T T T T . . . . . . . . . . . . . . . . . . . . . . . . . . . .    
83	. G G G G G G G G G G G G . . . . . . . . . . . . . . . . . . . . . . . . . . . .    
84	. G G G G G G G G G G G G . . . . . . . . . . . . . . . . . . . . . . . . . . . .    
85	. T T T T T T T T T T T T . . . . . . . . . . . . . . . . . . . . . . . . . . . .    
86	. C C C C C C C C C C C C C . . . . . . . . . . . . . . . . . . . . . . . . . . .    
87	. T T T T T T T T T T T T T . . . . . . . . . . . . . . . . . . . . . . . . . . .    
88	. C C C C C C C C C C C C C . . . . . . . . . . . . . . . . . . . . . . . . . . .    
89	. T T T T T T T T T T T T T . . . . . . . . . . . . . . . . . . . . . . . . . . .    
90	. G G G G G G G G G G G G G . . . . . . . . . . . . . . . . . . . . . . . . . . .    
91	. G G G G G G G G G G G G G . . . . . . . . . . . . . . . . . . . . . . . . . . .    
92	. G G G G G G G G G G G G G . . . . . . . . . . . . . . . . . . . . . . . . . . .    
93	. G G G G G G G G G G G G G . . . . . . . . . . . . . . . . . . . . . . . . . . .    
94	. T T T T T T T T T T T T T . . . . . . . . . . . . . . . . . . . . . . . . . . .    
95	. C C C C C C C C C C C C C . . . . . . . . . . . . . . . . . . . . . . . . . . .    
96	. A A A A A A A A A A A A A . . . . . . . . . . . . . . . . . . . . . . . . . . .    
97	. C C C C C C C C C C C C C . . . . . . . . . . . . . . . . . . . . . . . . . . .    
98	. C C C C C C C C C C C C C . . . . . . . . . . . . . . . . . . . . . . . . . . .    
99	. G G G G G G G A G G G G G . . . . . . . . . . . . . . . . . . . . . . . . . . .    
100	. G G G G G G G G G G G G G . . . . . . . . . . . . . . . . . . . . . . . . . . .    
101	. C C C C C C C C C C C C C . . . . . . . . . . . . . . . . . . . . . . . . . . .    
102	. A A A A A A A A A A A A A . . . . . . . . . . . . . . . . . . . . . . . . . . .    
103	. G G G G G G G G G G G G G . . . . . . . . . . . . . . . . . . . . . . . . . . .    
104	. T T T T T T T T T T T T T . . . . . . . . . . . . . . . . . . . . . . . . . . .    
105	. G G G G G G G G G G G G G . . . . . . . . . . . . . . . . . . . . . . . . . . .    
106	. T T T T T T T T T T T T T . . . . . . . . . . . . . . . . . . . . . . . . . . .    
107	. C C C C C C C C C C C C C . . . . . . . . . . . . . . . . . . . . . . . . . . .    
108	. T T T T T T T T T T T T T . . . . . . . . . . . . . . . . . . . . . . . . . . .    
109	. C C C C C C C C C C C C C . . . . . . . . . . . . . . . . . . . . . . . . . . .    
110	. T T T T T T T T T T T T T . . . . . . . . . . . . . . . . . . . . . . . . . . .    
111	. G G G G G G G G G G G G G . . . . . . . . . . . . . . . . . . . . . . . . . . .    
112	. T T T T T T T T T T T T T . . . . . . . . . . . . . . . . . . . . . . . . . . .    
113	. T T T T T T T T T T T T T . . . . . . . . . . . . . . . . . . . . . . . . . . .    
114	. G G G G G G G G G G G G G . . . . . . . . . . . . . . . . . . . . . . . . . . .    
115	. C C C C C C C C C C C C C . . . . . . . . . . . . . . . . . . . . . . . . . . .    
116	. C C C C C C C C C C C C C . . . . . . . . . . . . . . . . . . . . . . . . . . .    
117	. T T T T T T T T T T T T T . . . . . . . . . . . . . . . . . . . . . . . . . . .    
118	. T T T T T T T T T T T T T . . . . . . . . . . . . . . . . . . . . . . . . . . .    
119	. G G G G G G G G G G G G G G . . . . . . . . . . . . . . . . . . . . . . . . . .    
120	. C C C C C C C C C C C C C C . . . . . . . . . . . . . . . . . . . . . . . . . .    
121	. A A A A A A A A A A A A A A . . . . . . . . . . . . . . . . . . . . . . . . . .    
122	. T T T T T T T T T T T T T T . . . . . . . . . . . . . . . . . . . . . . . . . .    
123	. C C C C C C C C C C C C C C . . . . . . . . . . . . . . . . . . . . . . . . . .    
124	. C C C C C C C C C C C C C C . . . . . . . . . . . . . . . . . . . . . . . . . .    
125	. T T T T T T T T T T T T T T . . . . . . . . . . . . . . . . . . . . . . . . . .    
126	. C C C C C C C C C C C C C C . . . . . . . . . . . . . . . . . . . . . . . . . .    
127	. T T T T T T T T T T T T T T . . . . . . . . . . . . . . . . . . . . . . . . . .    
128	. C C C C C C C C C C C C C C . . . . . . . . . . . . . . . . . . . . . . . . . .    
129	. G G G G G G G G G G G G G G . . . . . . . . . . . . . . . . . . . . . . . . . .    
130	. T T T T T T T T T T T T T T . . . . . . . . . . . . . . . . . . . . . . . . . .    
131	. C C C C C C C C C C C C C C . . . . . . . . . . . . . . . . . . . . . . . . . .    
132	. A A A A A A A A A A A A A A . . . . . . . . . . . . . . . . . . . . . . . . . .    
133	. T T T T T T T T T T T T T T . . . . . . . . . . . . . . . . . . . . . . . . . .    
134	. C C C C C C C C C C C C C C . . . . . . . . . . . . . . . . . . . . . . . . . .    
135	. C C C C C C C C C C C C C C . . . . . . . . . . . . . . . . . . . . . . . . . .    
136	. T T T T T T T T T T T T T T . . . . . . . . . . . . . . . . . . . . . . . . . .    
137	. C C C C C C C C C C C C C C . . . . . . . . . . . . . . . . . . . . . . . . . .    
138	. A A A A A A A A A A A A A A . . . . . . . . . . . . . . . . . . . . . . . . . .    
139	. A A A A A A A A A A A A A A . . . . . . . . . . . . . . . . . . . . . . . . . .    
140	. C C C C C C C C C C C C C C . . . . . . . . . . . . . . . . . . . . . . . . . .    
141	. A A A A A A A A A A A A A A . . . . . . . . . . . . . . . . . . . . . . . . . .    
142	. T T T T T T T T T T T T T T . . . . . . . . . . . . . . . . . . . . . . . . . .    
143	. C C C C C C C C C C C C C C . . . . . . . . . . . . . . . . . . . . . . . . . .    
144	. T T T T T T T T T T T T T T . . . . . . . . . . . . . . . . . . . . . . . . . .    
145	. C C C C C C C C C C C C C C . . . . . . . . . . . . . . . . . . . . . . . . . .    
146	. A A A A A A A A A A A A A A . . . . . . . . . . . . . . . . . . . . . . . . . .    
147	. G G G G G G G G G G G G G G . . . . . . . . . . . . . . . . . . . . . . . . . .    
148	. A A A A A A A A A A A A A A . . . . . . . . . . . . . . . . . . . . . . . . . .    
149	. C C C C C C C C C C C C C C . . . . . . . . . . . . . . . . . . . . . . . . . .    
150	. C C C C C C C C C C C C C C . . . . . . . . . . . . . . . . . . . . . . . . . .    
151	. A A A A A A A A A A A A A A . . . . . . . . . . . . . . . . . . . . . . . . . .    
152	. C C C C C C C C C C C C C C . . . . . . . . . . . . . . . . . . . . . . . . . .    
153	. T T T T T T T T T T T T T T . . . . . . . . . . . . . . . . . . . . . . . . . .    
154	. G G G G G G G G G G G G G G . . . . . . . . . . . . . . . . . . . . . . . . . .    
155	. G G G G G G G G G G G G G G . . . . . . . . . . . . . . . . . . . . . . . . . .    
156	. A A A A A A A A A A A A A A . . . . . . . . . . . . . . . . . . . . . . . . . .    
157	. T T T T T T T T T T T T T T . . . . . . . . . . . . . . . . . . . . . . . . . .    
158	. T T T T T T T T T T T T T T . . . . . . . . . . . . . . . . . . . . . . . . . .    
159	. C C C C C C C C C C C C C C . . . . . . . . . . . . . . . . . . . . . . . . . .    
160	. G G G G G G G G G G G G G G . . . . . . . . . . . . . . . . . . . . . . . . . .    
161	. C C C C C C C C C C C C C C . . . . . . . . . . . . . . . . . . . . . . . . . .    
162	. A A A A A A A A A A A A A A . . . . . . . . . . . . . . . . . . . . . . . . . .    
163	. T T T T T T T T T T T T T T . . . . . . . . . . . . . . . . . . . . . . . . . .    
164	. C C C C C C C C C C C C C C . . . . . . . . . . . . . . . . . . . . . . . . . .    
165	. C C C C C C C C C C C C C C . . . . . . . . . . . . . . . . . . . . . . . . . .    
166	. G G G G G G G G G G G G G G . . . . . . . . . . . . . . . . . . . . . . . . . .    
167	. C C C C C C C C C C C C C C . . . . . . . . . . . . . . . . . . . . . . . . . .    
168	. T T T T T T T T T T T T T T . . . . . . . . . . . . . . . . . . . . . . . . . .    
169	. C C C C C C C C C C C C C C . . . . . . . . . . . . . . . . . . . . . . . . . .    
170	. C C C C C C C C C C C C C C . . . . . . . . . . . . . . . . . . . . . . . . . .    
171	. C C C C C C C C C C C C C C . . . . . . . . . . . . . . . . . . . . . . . . . .    
172	. A A A A A A A A A A A A A A . . . . . . . . . . . . . . . . . . . . . . . . . .    
173	. G G G G G G G G G G G G G G . . . . . . . . . . . . . . . . . . . . . . . . . .    
174	. G G G G G G G G G G G G G G . . . . . . . . . . . . . . . . . . . . . . . . . .    
175	. A A A A A A A A A A A A A A . . . . . . . . . . . . . . . . . . . . . . . . . .    
176	. G G G G G G G G G G G G G G . . . . . . . . . . . . . . . . . . . . . . . . . .    
177	. G G G G G G G G G G G G G G . . . . . . . . . . . . . . . . . . . . . . . . . .    
178	. G G G G G G G G G G G G G G . . . . . . . . . . . . . . . . . . . . . . . . . .    
179	. G G G G G G G G G G G G G G . . . . . . . . . . . . . . . . . . . . . . . . . .    
180	. C C C C C C C C C C C C C C . . . . . . . . . . . . . . . . . . . . . . . . . .    
181	. G G G G G G G G G G G G G G . . . . . . . . . . . . . . . . . . . . . . . . . .    
182	. G G G G G G G G G G G G G G . . . . . . . . . . . . . . . . . . . . . . . . . .    
183	. C C C C C C C C C C C C C C . . . . . . . . . . . . . . . . . . . . . . . . . .    
184	. C C C C C C C C C C C C C C . . . . . . . . . . . . . . . . . . . . . . . . . .    
185	. C C C C C C C C C C C C C C . . . . . . . . . . . . . . . . . . . . . . . . . .    
186	. A A A A A A A A A A A A A A . . . . . . . . . . . . . . . . . . . . . . . . . .    
187	. T T T T T T T T T T T T T T . . . . . . . . . . . . . . . . . . . . . . . . . .    
188	. G G G G G G G G G G G G G G . . . . . . . . . . . . . . . . . . . . . . . . . .    
189	. C C C C C C C C C C C C C C . . . . . . . . . . . . . . . . . . . . . . . . . .    
190	. A A A A A A A A A A A A A A . . . . . . . . . . . . . . . . . . . . . . . . . .    
191	. G G G G G G G G G G G G G G . . . . . . . . . . . . . . . . . . . . . . . . . .    
192	. G G G G G G G G G G G G G G . . . . . . . . . . . . . . . . . . . . . . . . . .    
193	. T T T T T T T T T T T T T T . . . . . . . . . . . . . . . . . . . . . . . . . .    
194	. G G G G G G G G G G G G G G . . . . . . . . . . . . . . . . . . . . . . . . . .    
195	. A A A A A A A A A A A A A A . . . . . . . . . . . . . . . . . . . . . . . . . .    
196	. T T T T T T T T T T T T T T . . . . . . . . . . . . . . . . . . . . . . . . . .    
197	. C C C C C C C C C C C C C C . . . . . . . . . . . . . . . . . . . . . . . . . .    
198	. G G G G G G G G G G G G G G . . . . . . . . . . . . . . . . . . . . . . . . . .    
199	. G G G G G G G G G G G G G G . . . . . . . . . . . . . . . . . . . . . . . . . .    
200	. C C C C C C C C C C C C C C . . . . . . . . . . . . . . . . . . . . . . . . . .    
201	. G G G G G G G G G G G G G G . . . . . . . . . . . . . . . . . . . . . . . . . .    
202	. C C C C C C C C C C C C C C . . . . . . . . . . . . . . . . . . . . . . . . . .    
203	. G G G G G G G G G G G G G G . . . . . . . . . . . . . . . . . . . . . . . . . .    
204	. T T T T T T T T T T T T T T . . . . . . . . . . . . . . . . . . . . . . . . . .    
205	. T T T T T T T T T T T T T T . . . . . . . . . . . . . . . . . . . . . . . . . .    
206	. A A A A A A A A A A A A A A . . . . . . . . . . . . . . . . . . . . . . . . . .    
207	. A A A A A A A A A A A A A A . . . . . . . . . . . . . . . . . . . . . . . . . .    
208	. T T T T T T T T T T T T T T . . . . . . . . . . . . . . . . . . . . . . . . . .    
209	. C C C C C C C C C C C C C C . . . . . . . . . . . . . . . . . . . . . . . . . .    
210	. G G G G G G G G G G G G G G . . . . . . . . . . . . . . . . . . . . . . . . . .    
211	. G G G G G G G G G G G G G G . . . . . . . . . . . . . . . . . . . . . . . . . .    
212	. G G G G G G G G G G G G G G . . . . . . . . . . . . . . . . . . . . . . . . . .    
213	. A A A A A A A A A A A A A A . . . . . . . . . . . . . . . . . . . . . . . . . .    
214	. A A A A A A A A A A A A A A . . . . . . . . . . . . . . . . . . . . . . . . . .    
215	. G G G G G G G G G G G G G G . . . . . . . . . . . . . . . . . . . . . . . . . .    
216	. C C C C C C C C C C C C C C . . . . . . . . . . . . . . . . . . . . . . . . . .    
217	. A A A A A A A A A A A A A A . . . . . . . . . . . . . . . . . . . . . . . . . .    
218	. G G G G G G G G G G G G G G . . . . . . . . . . . . . . . . . . . . . . . . . .    
219	. G G G G G G G G G G G G G G . . . . . . . . . . . . . . . . . . . . . . . . . .    
220	. A A A A A A A A A A A A A A . . . . . . . . . . . . . . . . . . . . . . . . . .    
221	. G G G G G G G G G G G G G G . . . . . . . . . . . . . . . . . . . . . . . . . .    
222	. G G G G G G G G G G G G G G . . . . . . . . . . . . . . . . . . . . . . . . . .    
223	. G G G G G G G G G G G G G G . . . . . . . . . . . . . . . . . . . . . . . . . .    
224	. T T T T T T T T T T T T T T . . . . . . . . . . . . . . . . . . . . . . . . . .    
225	. A A A A A A A A A A A A A A . . . . . . . . . . . . . . . . . . . . . . . . . .    
226	. G G G G G G G G G G G G G G . . . . . . . . . . . . . . . . . . . . . . . . . .    
227	. A A A A A A A A A A A A A A . . . . . . . . . . . . . . . . . . . . . . . . . .    
228	. A A A A A A A A A A A A A A . . . . . . . . . . . . . . . . . . . . . . . . . .    
229	. A A A A A A A A A A A A A A . . . . . . . . . . . . . . . . . . . . . . . . . .    
230	. C C C C C C C C C C C C C C . . . . . . . . . . . . . . . . . . . . . . . . . .    
231	. A A A A A A A A A A A A A A . . . . . . . . . . . . . . . . . . . . . . . . . .    
232	. T T T T T T T T T T T T T T . . . . . . . . . . . . . . . . . . . . . . . . . .    
233	. T T T T T T T T T T T T T T . . . . . . . . . . . . . . . . . . . . . . . . . .    
234	. G G G G G G G G G G G G G G . . . . . . . . . . . . . . . . . . . . . . . . . .    
235	. A A A A A A A A A A A A A A . . . . . . . . . . . . . . . . . . . . . . . . . .    
236	. G G G G G G G G G G G G G G . . . . . . . . . . . . . . . . . . . . . . . . . .    
237	. G G G G G G G G G G G G G G . . . . . . . . . . . . . . . . . . . . . . . . . .    
238	. T T T T T T T T T T T T T T . . . . . . . . . . . . . . . . . . . . . . . . . .    
239	. G G G G G G G G G G G G G G . . . . . . . . . . . . . . . . . . . . . . . . . .    
240	. A A A A A A A A A A A A A A . . . . . . . . . . . . . . . . . . . . . . . . . .    
241	. T T T T T T T T T T T T T T . . . . . . . . . . . . . . . . . . . . . . . . . .    
242	. G G G G G G G G G G G G G G . . . . . . . . . . . . . . . . . . . . . . . . . .    
243	. A A A A A A A A A A A A A A . . . . . . . . . . . . . . . . . . . . . . . . . .    
244	. A A A A A A A A A A A A A A . . . . . . . . . . . . . . . . . . . . . . . . . .    
245	. C C C C C C C C C C C C C C . . . . . . . . . . . . . . . . . . . . . . . . . .    
246	. T T T T T T T T T T T T T T . . . . . . . . . . . . . . . . . . . . . . . . . .    
247	. C C C C C C C C C C C C C C . . . . . . . . . . . . . . . . . . . . . . . . . .    
248	. C C C C C C C C C C C C C C . . . . . . . . . . . . . . . . . . . . . . . . . .    
249	. T T T T T T T T T T T T T T . . . . . . . . . . . . . . . . . . . . . . . . . .    
250	. T T T T T T T T T T T T T T . . . . . . . . . . . . . . . . . . . . . . . . . .    
251	. T T T T T T T T T T T T T T . . . . . . . . . . . . . . . . . . . . . . . . . .    
252	. G G G G G G G G G G G G G G . . . . . . . . . . . . . . . . . . . . . . . . . .    
253	. A A A A A A A A A A A A A A . . . . . . . . . . . . . . . . . . . . . . . . . .    
254	. G G G G G G G G G G G G G G . . . . . . . . . . . . . . . . . . . . . . . . . .    
255	. C C C C C C C C C C C C C C . . . . . . . . . . . . . . . . . . . . . . . . . .    
256	. T T T T T T T T T T T T T T . . . . . . . . . . . . . . . . . . . . . . . . . .    
257	. G G G G G G G G G G G G G G . . . . . . . . . . . . . . . . . . . . . . . . . .    
258	. C C C C C C C C C C C C C C . . . . . . . . . . . . . . . . . . . . . . . . . .    
259	. T T T T T T T T T T T T T T . . . . . . . . . . . . . . . . . . . . . . . . . .    
260	. T T T T T T T T T T T T T T . . . . . . . . . . . . . . . . . . . . . . . . . .    
261	. C C C C C C C C C C C C C C . . . . . . . . . . . . . . . . . . . . . . . . . .    
262	. A A A A A A A A A A A A A A . . . . . . . . . . . . . . . . . . . . . . . . . .    
263	. T T T T T T T T T T T T T T . . . . . . . . . . . . . . . . . . . . . . . . . .    
264	. C C C C C C C C C C C C C C . . . . . . . . . . . . . . . . . . . . . . . . . .    
265	. A A A A A A A A A A A A A A . . . . . . . . . . . . . . . . . . . . . . . . . .    
266	. A A A A A A A A A A A A A A . . . . . . . . . . . . . . . . . . . . . . . . . .    
267	. T T T T T T T T T T T T T T . . . . . . . . . . . . . . . . . . . . . . . . . .    
268	. T T T T T T T T T T T T T T . . . . . . . . . . . . . . . . . . . . . . . . . .    
269	. G G G G G G G G G G G G G G . . . . . . . . . . . . . . . . . . . . . . . . . .    
270	. G G G G G G G G G G G G G G . . . . . . . . . . . . . . . . . . . . . . . . . .    
271	. T T T T T T T T T T T T T T . . . . . . . . . . . . . . . . . . . . . . . . . .    
272	. A A A A A A A A A A A A A A . . . . . . . . . . . . . . . . . . . . . . . . . .    
273	. G G G G G G G G G G G G G G . . . . . . . . . . . . . . . . . . . . . . . . . .    
274	. A A A A A A A A A A A A A A . . . . . . . . . . . . . . . . . . . . . . . . . .    
275	. T T T T T T T T T T T T T T . . . . . . . . . . . . . . . . . . . . . . . . . .    
276	. G G G G G G G G G G G G G G . . . . . . . . . . . . . . . . . . . . . . . . . .    
277	. A A A A A A A A A A A A A A . . . . . . . . . . . . . . . . . . . . . . . . . .    
278	. C C C C C C C C C C C C C C . . . . . . . . . . . . . . . . . . . . . . . . . .    
279	. C C C C C C C C C C C C C C . . . . . . . . . . . . . . . . . . . . . . . . . .    
280	. G G G G G G G G G G G G G G . . . . . . . . . . . . . . . . . . . . . . . . . .    
281	. A A A A A A A A A A A A A A . . . . . . . . . . . . . . . . . . . . . . . . . .    
282	. G G G G G G G G G G G G G G . . . . . . . . . . . . . . . . . . . . . . . . . .    
283	. C C C C C C C C C C C C C C . . . . . . . . . . . . . . . . . . . . . . . . . .    
284	. A A A A A A A A A A A A A A . . . . . . . . . . . . . . . . . . . . . . . . . .    
285	. C C C C C C C C C C C C C C . . . . . . . . . . . . . . . . . . . . . . . . . .    
286	. A A A A A A A A A A A A A A . . . . . . . . . . . . . . . . . . . . . . . . . .    
287	. C C C C C C C C C C C C C C . . . . . . . . . . . . . . . . . . . . . . . . . .    
288	. A A A A A A A A A A A A A A . . . . . . . . . . . . . . . . . . . . . . . . . .    
289	. T T T T T T T T T T T T T T . . . . . . . . . . . . . . . . . . . . . . . . . .    
290	. T T T T T T T T T T T T T T . . . . . . . . . . . . . . . . . . . . . . . . . .    
291	. G G G G G G G G G G G G G G . . . . . . . . . . . . . . . . . . . . . . . . . .    
292	. A A A A G A A A A A A A A A . . . . . . . . . . . . . . . . . . . . . . . . . .    
293	. C C C C C C C C C C C C C C . . . . . . . . . . . . . . . . . . . . . . . . . .    
294	. A A A A A A A A A A A A A A . . . . . . . . . . . . . . . . . . . . . . . . . .    
295	. A A A A A A A A A A A A A A . . . . . . . . . . . . . . . . . . . . . . . . . .    
296	. G G G G G G G G G G G G G G . . . . . . . . . . . . . . . . . . . . . . . . . .    
297	. G G G G G G G G G G G G G G . . . . . . . . . . . . . . . . . . . . . . . . . .    
298	. A A A A A A A A A A A A A A . . . . . . . . . . . . . . . . . . . . . . . . . .    
299	. G G G G G G G G G G G G G G . . . . . . . . . . . . . . . . . . . . . . . . . .    
300	. T T T T T T T T T T T T T T . . . . . . . . . . . . . . . . . . . . . . . . . .    
301	. A A A A A A A A A A A A A A . . . . . . . . . . . . . . . . . . . . . . . . . .    
302	. T T T T T T T T T T T T T T . . . . . . . . . . . . . . . . . . . . . . . . . .    
303	. T T T T T T T T T T T T T T . . . . . . . . . . . . . . . . . . . . . . . . . .    
304	. A A A A A A A A A A A A A A . . . . . . . . . . . . . . . . . . . . . . . . . .    
305	. C C C C C C C C C C C C C C . . . . . . . . . . . . . . . . . . . . . . . . . .    
306	. T T T T T T T T T T T T T T . . . . . . . . . . . . . . . . . . . . . . . . . .    
307	. A A A A A A A A A A A A A A . . . . . . . . . . . . . . . . . . . . . . . . . .    
308	. C C C C C C C C C C C C C C . . . . . . . . . . . . . . . . . . . . . . . . . .    
309	. A A A A A A A A A A A A A A . . . . . . . . . . . . . . . . . . . . . . . . . .    
310	. C C C C C C C C C C C C C C . . . . . . . . . . . . . . . . . . . . . . . . . .    
311	. C C C C C C C C C C C C C C . . . . . . . . . . . . . . . . . . . . . . . . . .    
312	. A A A A A A A A A A A A A A . . . . . . . . . . . . . . . . . . . . . . . . . .    
313	. A A A A A A A A A A A A A A . . . . . . . . . . . . . . . . . . . . . . . . . .    
314	. G G G G G G G G G G G G G G . . . . . . . . . . . . . . . . . . . . . . . . . .    
315	. G G G G G G G G G G G G G G . . . . . . . . . . . . . . . . . . . . . . . . . .    
316	. A A A A A A A A A A A A A A . . . . . . . . . . . . . . . . . . . . . . . . . .    
317	. G G G G G G G G G G G G G G . . . . . . . . . . . . . . . . . . . . . . . . . .    
318	. G G G G G G G G G G G G G G . . . . . . . . . . . . . . . . . . . . . . . . . .    
319	. A A A A A A A A A A A A A A . . . . . . . . . . . . . . . . . . . . . . . . . .    
320	. G G G G G G G G G G G G G G . . . . . . . . . . . . . . . . . . . . . . . . . .    
321	. C C C C C C C C C C C C C C . . . . . . . . . . . . . . . . . . . . . . . . . .    
322	. A A A A A A A A A A A A A A . . . . . . . . . . . . . . . . . . . . . . . . . .    
323	. G G G G G G G G G G G G G G . . . . . . . . . . . . . . . . . . . . . . . . . .    
324	. T T T T T T T T T T T T T T . . . . . . . . . . . . . . . . . . . . . . . . . .    
325	. T T T T T T T T T T T T T T . T . . . . . . . . . . . . . . . . . . . . . . . .    
326	. C C C C C C C C C C C C C C . C . . . . . . . . . . . . . . . . . . . . . . . .    
327	. A A A A A A A A A A A A A A . A . . . . . . . . . . . . . . . . . . . . . . . .    
328	. A A A A A A A A A A A A A A . A . . . . . . . . . . . . . . . . . . . . . . . .    
329	. A A A A A A A A A A A A A A . A . . . . . . . . . . . . . . . . . . . . . . . .    
330	. C C C C C C C C C C C C C C . C . . . . . . . . . . . . . . . . . . . . . . . .    
331	. A A A A A A A A A A A A A A . A . . . . . . . . . . . . . . . . . . . . . . . .    
332	. G G G G G G G G G G G G G G . G . . . . . . . . . . . . . . . . . . . . . . . .    
333	. G G G G G G G G G G G G G G . G . . . . . . . . . . . . . . . . . . . . . . . .    
334	. T T T T T T T T T T T T T T . T . . . . . . . . . . . . . . . . . . . . . . . .    
335	. C C C C C C C C C C C C C C . C . . . . . . . . . . . . . . . . . . . . . . . .    
336	. T T T T T T T T T T T T T T . T . . . . . . . . . . . . . . . . . . . . . . . .    
337	. T T T T T T T T T T T T T T . T . . . . . . . . . . . . . . . . . . . . . . . .    
338	. C C C C C C C C C C C C C C . C . . . . . . . . . . . . . . . . . . . . . . . .    
339	. A A A A A A A A A A A A A A . A . . . . . . . . . . . . . . . . . . . . . . . .    
340	. A A A A A A A A A A A A A A . A . . . . . . . . . . . . . . . . . . . . . . . .    
341	. G G G G G G G G G G G G G G . G . . . . . . . . . . . . . . . . . . . . . . . .    
342	. G G G G G G G G G G G G G G . G . . . . . . . . . . . . . . . . . . . . . . . .    
343	. A A A A A A A A A A A A A A . A . . . . . . . . . . . . . . . . . . . . . . . .    
344	. C C C C C C C C C C C C C C . C . . . . . . . . . . . . . . . . . . . . . . . .    
345	. A A A A A A A A A A A A A A . A . . . . . . . . . . . . . . . . . . . . . . . .    
346	. T T T T T T T T T T T T T T . T . . . . . . . . . . . . . . . . . . . . . . . .    
347	. G G G G G G G G G G G G G G G G . . . . . . . . . . . . . . . . . . . . . . . .    
348	. G G G G G G G G G G G G G G G G . . . . . . . . . . . . . . . . . . . . . . . .    
349	. A A A A A A A A A A A A A A A A . . . . . . . . . . . . . . . . . . . . . . . .    
350	. A A A A A A A A A A A A A A A A . . . . . . . . . . . . . . . . . . . . . . . .    
351	. T T T T T T T T T T T T T T T T . . . . . . . . . . . . . . . . . . . . . . . .    
352	. T T T T T T T T T T T T T T T T . . . . . . . . . . . . . . . . . . . . . . . .    
353	. C C C C C C C C C C C C C C C C . . . . . . . . . . . . . . . . . . . . . . . .    
354	. C C C C C C C C C C C C C C C C . . . . . . . . . . . . . . . . . . . . . . . .    
355	. T T T T T T T T T T T T T T T T . . . . . . . . . . . . . . . . . . . . . . . .    
356	. G G G G G G G G G G G G G G G G . . . . . . . . . . . . . . . . . . . . . . . .    
357	. G G G G G G G G G G G G G G G G . . . . . . . . . . . . . . . . . . . . . . . .    
358	. G G G G G G G G G G G G G G G G . . . . . . . . . . . . . . . . . . . . . . . .    
359	. C C C C C C C C C C C C C C C C . . . . . . . . . . . . . . . . . . . . . . . .    
360	. T T T T T T T T T T T T T T T T . . . . . . . . . . . . . . . . . . . . . . . .    
361	. G G G G G G G G G G G G G G G G . . . . . . . . . . . . . . . . . . . . . . . .    
362	. G G G G G G G G G G G G G G G G . . . . . . . . . . . . . . . . . . . . . . . .    
363	. T T T T T T T T T T T T T T T T . . . . . . . . . . . . . . . . . . . . . . . .    
364	. A A A A A A A A A A A A A A A A . . . . . . . . . . . . . . . . . . . . . . . .    
365	. T T T T T T T T T T T T T T T T . . . . . . . . . . . . . . . . . . . . . . . .    
366	. A A A A A A A A A A A A A A A A . . . . . . . . . . . . . . . . . . . . . . . .    
367	. C C C C C C C C C C C C C C C C . . . . . . . . . . . . . . . . . . . . . . . .    
368	. C C C C C C C C C C C C C C C C . . . . . . . . . . . . . . . . . . . . . . . .    
369	. A A A A A A A A A A A A A A A A . . . . . . . . . . . . . . . . . . . . . . . .    
370	. C C C C C C C C C C C C C C C C . . . . . . . . . . . . . . . . . . . . . . . .    
371	. A A A A A A A A A A A A A A A A . . . . . . . . . . . . . . . . . . . . . . . .    
372	. G G G G G G G G G G G G G G G G . . . . . . . . . . . . . . . . . . . . . . . .    
373	. G G G G G G G G G G G G G G G G . . . . . . . . . . . . . . . . . . . . . . . .    
374	. T T T T T T T T T T T T T T T T . . . . . . . . . . . . . . . . . . . . . . . .    
375	. G G G G G G G G G G G G G G G G . . . . . . . . . . . . . . . . . . . . . . . .    
376	. G G G G G G G G G G G G G G G G . . . . . . . . . . . . . . . . . . . . . . . .    
377	. T T T T T T T T T T T T T T T T . . . . . . . . . . . . . . . . . . . . . . . .    
378	. C C C C C C C C C C C C C C C C . . . . . . . . . . . . . . . . . . . . . . . .    
379	. C C C C C C C C C C C C C C C C . . . . . . . . . . . . . . . . . . . . . . . .    
380	. T T T T T T T T T T T T T T T T . . . . . . . . . . . . . . . . . . . . . . . .    
381	. T T T T T T T T T T T T T T T T . . . . . . . . . . . . . . . . . . . . . . . .    
382	. G G G G G G G G G G G G G G G G . . . . . . . . . . . . . . . . . . . . . . . .    
383	. T T T T T T T T T T T T T T T T . . . . . . . . . . . . . . . . . . . . . . . .    
384	. G G G G G G G G G G G G G G G G . . . . . . . . . . . . . . . . . . . . . . . .    
385	. A A A A A A A A A A A A A A A A . . . . . . . . . . . . . . . . . . . . . . . .    
386	. C C C C C C C C C C C C C C C C . . . . . . . . . . . . . . . . . . . . . . . .    
387	. C C C C C C C C C C C C C C C C . . . . . . . . . . . . . . . . . . . . . . . .    
388	. A A A A A A A A A A A A A A A A . . . . . . . . . . . . . . . . . . . . . . . .    
389	. A A A A A A A A A A A A A A A A . . . . . . . . . . . . . . . . . . . . . . . .    
390	. T T T T T T T T T T T T T T T T . . . . . . . . . . . . . . . . . . . . . . . .    
391	. C C C C C C C C C C C C C C C C . . . . . . . . . . . . . . . . . . . . . . . .    
392	. A A A A A A A A A A A A A A A A . . . . . . . . . . . . . . . . . . . . . . . .    
393	. G G G G G G G G G G G G G G G G . . . . . . . . . . . . . . . . . . . . . . . .    
394	. A A A A A A A A A A A A A A A A . . . . . . . . . . . . . . . . . . . . . . . .    
395	. C C C C C C C C C C C C C C C C . . . . . . . . . . . . . . . . . . . . . . . .    
396	. A A A A A A A A A A A A A A A A . . . . . . . . . . . . . . . . . . . . . . . .    
397	. T T T T T T T T T T T T T T T T . . . . . . . . . . . . . . . . . . . . . . . .    
398	. C C C C C C C C C C C C C C C C . . . . . . . . . . . . . . . . . . . . . . . .    
399	. C C C C C C C C C C C C C C C C . . . . . . . . . . . . . . . . . . . . . . . .    
400	. A A A A A A A A A A A A A A A A . . . . . . . . . . . . . . . . . . . . . . . .    
401	. C C C C C C C C C C C C C C C C . . . . . . . . . . . . . . . . . . . . . . . .    
402	. A A A A A A A A A A A A A A A A . . . . . . . . . . . . . . . . . . . . . . . .    
403	. T T T T T T T T T T T T T T T T . . . . . . . . . . . . . . . . . . . . . . . .    
404	. T T T T T T T T T T T T T T T T . . . . . . . . . . . . . . . . . . . . . . . .    
405	. C C C C C C C C C C C C C C C C . . . . . . . . . . . . . . . . . . . . . . . .    
406	. A A A A A A A A A A A A A A A A . . . . . . . . . . . . . . . . . . . . . . . .    
407	. C C C C C C C C C C C C C C C C . . . . . . . . . . . . . . . . . . . . . . . .    
408	. A A A A A A A A A A A A A A A A . . . . . . . . . . . . . . . . . . . . . . . .    
409	. A A A A A A A A A A A A A A A A . . . . . . . . . . . . . . . . . . . . . . . .    
410	. G G G G G G G G G G G G G G G G . . . . . . . . . . . . . . . . . . . . . . . .    
411	. C C C C C C C C C C C C C C C C . . . . . . . . . . . . . . . . . . . . . . . .    
412	. A A A A A A A A A A A A A A A A . . . . . . . . . . . . . . . . . . . . . . . .    
413	. G G G G G G G G G G G G G G G G . . . . . . . . . . . . . . . . . . . . . . . .    
414	. G G G G G G G G G G G G G G G G . . . . . . . . . . . . . . . . . . . . . . . .    
415	. T T T T T T T T T T T T T T T T . . . . . . . . . . . . . . . . . . . . . . . .    
416	. G G G G G G G G G G G G G G G G . . . . . . . . . . . . . . . . . . . . . . . .    
417	. T T T T T T T T T T T T T T T T . . . . . . . . . . . . . . . . . . . . . . . .    
418	. G G G G G G G G G G G G G G G G . . . . . . . . . . . . . . . . . . . . . . . .    
419	. T T T T T T T T T T T T T T T T . . . . . . . . . . . . . . . . . . . . . . . .    
420	. G G G G G G G G G G G G G G G G . . . . . . . . . . . . . . . . . . . . . . . .    
421	. A A A A A A A A A A A A A A A A . . . . . . . . . . . . . . . . . . . . . . . .    
422	. G G G G G G G G G G G G G G G G . . . . . . . . . . . . . . . . . . . . . . . .    
423	. A A A G A A A A A A A A A A A A . . . . . . . . . . . . . . . . . . . . . . . .    
424	. T T T T T T T T T T T T T T T T . . . . . . . . . . . . . . . . . . . . . . . .    
425	. A A A A A A A A A A A A A A A A . . . . . . . . . . . . . . . . . . . . . . . .    
426	. A A A A A A A A A A A A A A A A . . . . . . . . . . . . . . . . . . . . . . . .    
427	. T T T T T T T T T T T T T T T T . . . . . . . . . . . . . . . . . . . . . . . .    
428	. T T T T T T T T T T T T T T T T . . . . . . . . . . . . . . . . . . . . . . . .    
429	. G G G G G G G G G G G G G G G G . . . . . . . . . . . . . . . . . . . . . . . .    
430	. A A A A A A A A A A A A A A A A . . . . . . . . . . . . . . . . . . . . . . . .    
431	. G G G G G G G G G G G G G G G G . . . . . . . . . . . . . . . . . . . . . . . .    
432	. A A A A A A A A A A A A A A A A . . . . . . . . . . . . . . . . . . . . . . . .    
433	. G G G G G G G G G G G G G G G G . . . . . . . . . . . . . . . . . . . . . . . .    
434	. T T T T T T T T T T T T T T T T . . . . . . . . . . . . . . . . . . . . . . . .    
435	. C C C C C C C C C C C C C C C C . . . . . . . . . . . . . . . . . . . . . . . .    
436	. C C C C C C C C C C C C C C C C . . . . . . . . . . . . . . . . . . . . . . . .    
437	. T T T T T T T T T T T T T T T T . . . . . . . . . . . . . . . . . . . . . . . .    
438	. C C C C C C C C C C C C C C C C . . . . . . . . . . . . . . . . . . . . . . . .    
439	. T T T T T T T T T T T T T T T T . . . . . . . . . . . . . . . . . . . . . . . .    
440	. C C C C C C C C C C C C C C C C . . . . . . . . . . . . . . . . . . . . . . . .    
441	. T T T T T T T T T T T T T T T T . . . . . . . . . . . . . . . . . . . . . . . .    
442	T T T T T T T T T T T T T T T T T . . . . . . . . . . . . . . . . . . . . . . . .    
443	C C C C C C C C C C C C C C C C C . . . . . . . . . . . . . . . . . . . . . . . .    
444	C C C C C C C C C C C C C C C C C . . . . . . . . . . . . . . . . . . . . . . . .    
445	T T T T T T T T T T T T T T T T T . . . . . . . . . . . . . . . . . . . . . . . .    
446	C C C C C C C C C C C C C C C C C . . . . . . . . . . . . . . . . . . . . . . . .    
447	A A A A A A A A A A A A A A A A A . . . . . . . . . . . . . . . . . . . . . . . .    
448	A A A A A A A A A A A A A A A A A . . . . . . . . . . . . . . . . . . . . . . . .    
449	G G G G G G G G G G G G G G G G G . . . . . . . . . . . . . . . . . . . . . . . .    
450	C C C C C C C C C C C C C C C C C . . . . . . . . . . . . . . . . . . . . . . . .    
451	T T T T T T T T T T T T T T T T T . . . . . . . . . . . . . . . . . . . . . . . .    
452	C C C C C C C C C C C C C C C C C . . . . . . . . . . . . . . . . . . . . . . . .    
453	A A A A A A A A A A A A A A A A A . . . . . . . . . . . . . . . . . . . . . . . .    
454	A A A A A A A A A A A A A A A A A . . . . . . . . . . . . . . . . . . . . . . . .    
455	C C C C C C C C C C C C C C C C C . . . . . . . . . . . . . . . . . . . . . . . .    
456	C C C C C C C C C C C C C C C C C . . . . . . . . . . . . . . . . . . . . . . . .    
457	C C C C C C C C C C C C C C C C C . . . . . . . . . . . . . . . . . . . . . . . .    
458	G G G G G G G G G G G G G G G G G . . . . . . . . . . . . . . . . . . . . . . . .    
459	A A A A A A A A A A A A A A A A A . . . . . . . . . . . . . . . . . . . . . . . .    
460	T T T T T T T T T T T T T T T T T . . . . . . . . . . . . . . . . . . . . . . . .    
461	G G G G G G G G G G G G G G G G G . . . . . . . . . . . . . . . . . . . . . . . .    
462	A A A A A A A A A A A A A A A A A . . . . . . . . . . . . . . . . . . . . . . . .    
463	C C C C C C C C C C C C C C C C C . . . . . . . . . . . . . . . . . . . . . . . .    
464	C C C C C C C C C C C C C C C C C . . . . . . . . . . . . . . . . . . . . . . . .    
465	A A A A A A A A A A A A A A A A A . . . . . . . . . . . . . . . . . . . . . . . .    
466	A A A A A A A A A A A A A A A A A . . . . . . . . . . . . . . . . . . . . . . . .    
467	A A A A A A A A A A A A A A A A A . . . . . . . . . . . . . . . . . . . . . . . .    
468	C C C C C C C C C C C C C C C C C . . . . . . . . . . . . . . . . . . . . . . . .    
469	A A A A A A A A A A A A A A A A A . . . . . . . . . . . . . . . . . . . . . . . .    
470	C C C C C C C C C C C C C C C C C . . . . . . . . . . . . . . . . . . . . . . . .    
471	A A A A A A A A A A A A A A A A A . . . . . . . . . . . . . . . . . . . . . . . .    
472	C C C C C C C C C C C C C C C C C . . . . . . . . . . . . . . . . . . . . . . . .    
473	A A A A A A A A A A A A A A A A A . . . . . . . . . . . . . . . . . . . . . . . .    
474	G G G G G G G G G G G G G G G G G . . . . . . . . . . . . . . . . . . . . . . . .    
475	A A A A A A A A A A A A A A A A A . . . . . . . . . . . . . . . . . . . . . . . .    
476	T T T T T T T T T T T T T T T T T . . . . . . . . . . . . . . . . . . . . . . . .    
477	C C C C C C C C C C C C C C C C C . . . . . . . . . . . . . . . . . . . . . . . .    
478	T T T T T T T T T T T T T T T T T . . . . . . . . . . . . . . . . . . . . . . . .    
479	C C C C C C C C C C C C C C C C C . . . . . . . . . . . . . . . . . . . . . . . .    
480	C C C C C C C C C C C C C C C C C . . . . . . . . . . . . . . . . . . . . . . . .    
481	C C C C C C C C C C C C C C C C C . . . . . . . . . . . . . . . . . . . . . . . .    
482	T T T T T T T T T T T T T T T T T . . . . . . . . . . . . . . . . . . . . . . . .    
483	G G G G G G G G G G G G G G G G G . . . . . . . . . . . . . . . . . . . . . . . .    
484	T T T T T T T T T T T T T T T T T . . . . . . . . . . . . . . . . . . . . . . . .    
485	C C C C C C C C C C C C C C C C C . . . . . . . . . . . . . . . . . . . . . . . .    
486	A A A A A A A A A A A A A A A A A . . . . . . . . . . . . . . . . . . . . . . . .    
487	G G G G G G G G G G G G G G G G G . . . . . . . . . . . . . . . . . . . . . . . .    
488	T T T T T T T T T T T T T T T T T . . . . . . . . . . . . . . . . . . . . . . . .    
489	G G G G G G G G G G G G G G G G G . . . . . . . . . . . . . . . . . . . . . . . .    
490	T T T T T T T T T T T T T T T T T . . . . . . . . . . . . . . . . . . . . . . . .    
491	G G G G G G G G G G G G G G G G G . . . . . . . . . . . . . . . . . . . . . . . .    
492	T T T T T T T T T T T T T T T T T . . . . . . . . . . . . . . . . . . . . . . . .    
493	A A A A A A A A A A A A A A A A A . . . . . . . . . . . . . . . . . . . . . . . .    
494	T T T T T T T T T T T T T T T T T . . . . . . . . . . . . . . . . . . . . . . . .    
495	G G G G G G G G G G G G G G G G G . . . . . . . . . . . . . . . . . . . . . . . .    
496	A A A A A A A A A A A A A A A A A . . . . . . . . . . . . . . . . . . . . . . . .    
497	G G G G G G G G G G G G G G G G G . . . . . . . . . . . . . . . . . . . . . . . .    
498	T T T T T T T T T T T T T T T T T . . . . . . . . . . . . . . . . . . . . . . . .    
499	C C C C C C C C C C C C C C C C C . . . . . . . . . . . . . . . . . . . . . . . .    
500	T T T T T T T T T T T T T T T T T . . . . . . . . . . . . . . . . . . . . . . . .    
501	G G G G G G G G G G G G G G G G G . . . . . . . . . . . . . . . . . . . . . . . .    
502	T T T T T T T T T T T T T T T T T . . . . . . . . . . . . . . . . . . . . . . . .    
503	C C C C C C C C C C C C C C C C C . . . . . . . . . . . . . . . . . . . . . . . .    
504	A A A A A A A A A A A A A A A A A . . . . . . . . . . . . . . . . . . . . . . . .    
505	T T T T T T T T T T T T T T T T T . . . . . . . . . . . . . . . . . . . . . . . .    
506	C C C C C C C C C C C C C C C C C . . . . . . . . . . . . . . . . . . . . . . . .    
507	G G G G G G G G G G G G G G G G G . . . . . . . . . . . . . . . . . . . . . . . .    
508	A A A A A A A A A A A A A A A A A . . . . . . . . . . . . . . . . . . . . . . . .    
509	C C C C C C C C C C C C C C C C C . . . . . . . . . . . . . . . . . . . . . . . .    
510	A A A A A A A A A A A A A A A A A . . . . . . . . . . . . . . . . . . . . . . . .    
511	T T T T T T T T T T T T T T T T T . . . . . . . . . . . . . . . . . . . . . . . .    
512	C C C C C C C C C C C C C C C C C . . . . . . . . . . . . . . . . . . . . . . . .    
513	A A A A A A A A A A A A A A A A A . . . . . . . . . . . . . . . . . . . . . . . .    
514	T T T T T T T T T T T T T T T T T . . . T . . . . . . . . . . . . . . . . . . . .    
515	C C C C C C C C C C C C C C C C C . . . C . . . . . . . . . . . . . . . . . . . .    
516	A A A A A A A A A A A A A A A A A . . . A . . . . . . . . . . . . . . . . . . . .    
517	G G G G G G G G G G G G G G G G G . . . G . . . . . . . . . . . . . . . . . . . .    
518	T T T T T T T T T T T T T T T T T . . . T . . . . . . . . . . . . . . . . . . . .    
519	G G G G G G G G G G G G G G G G G . . . G . . . . . . . . . . . . . . . . . . . .    
520	G G G G G G G G G G G G G G G G G . . . G . . . . . . . . . . . . . . . . . . . .    
521	A A A A A A A A A A A A A A A A A . . . A . . . . . . . . . . . . . . . . . . . .    
522	G G G G G G G G G G G G G G G G G . . . G . . . . . . . . . . . . . . . . . . . .    
523	A A A A A A A A A A A A A A A A A . . . A . . . . . . . . . . . . . . . . . . . .    
524	G G G G G G G G G G G G G G G G G . . . G . . . . . . . . . . . . . . . . . . . .    
525	G G G G G G G G G G G G G G G G G . . . G . . . . . . . . . . . . . . . . . . . .    
526	T C C C C C C C C C C C C C C C C . . . C . . . . . . . . . . . . . . . . . . . .    
527	. G G G G G G G G G G G G G G G G . . . G . . . . . . . . . . . . . . . . . . . .    
528	. A A A A A A A A A A A A A A A A . . . A . . . . . . . . . . . . . . . . . . . .    
529	. C C C C C C C C C C C C C C C C . . . C . . . . . . . . . . . . . . . . . . . .    
530	. C C C C C C C C C C C C C C C C . . . C . . . . . . . . . . . . . . . . . . . .    
531	. A A A A A A A A A A A A A A A A . . . A . A . . . . . . . . . . . . . . . . . .    
532	. T T T T T T T T T T T T T T T T . . . T . T . . . . . . . . . . . . . . . . . .    
533	. G G G G G G G G G G G G G G G G . . . G . G . . . . . . . . . . . . . . . . . .    
534	. T T T T T T T T T T T T T T T T . . . T . T . . . . . . . . . . . . . . . . . .    
535	. T T T T T T T T T T T T T T T T . . . T . T . . . . . . . . . . . . . . . . . .    
536	. G G G G G G G G G G G G G G G G . . . G . G . . . . . . . . . . . . . . . . . .    
537	. T T T T T T T T T T T T T T T T . . . T . T . . . . . . . . . . . . . . . . . .    
538	. T T T T T T T T T T T T T T T T . . . T . T . . . . . . . . . . . . . . . . . .    
539	. T T T T T T T T T T T T T T T T . . . T . T . . . . . . . . . . . . . . . . . .    
540	. G G G G G G G G G G G G G G G G . . . G . G . . . . . . . . . . . . . . . . . .    
541	. C C C C C C C C C C C C C C C C . . . C . C . . . . . . . . . . . . . . . . . .    
542	. T T T T T T T T T T T T T T T T . . . T . T . . . . . . . . . . . . . . . . . .    
543	. G G G G G G G G G G G G G G G G . . . G . G . . . . . . . . . . . . . . . . . .    
544	. A A A A A A A A A A A A A A A A . . . A . A . . . . . . . . . . . . . . . . . .    
545	. G G G G G G G G G G G G G G G G . . . G . G . . . . . . . . . . . . . . . . . .    
546	. C C C C C C C C C C C C C C C C . . . C . C . . . . . . . . . . . . . . . . . .    
547	. T T T T T T T T T T T T T T T T . . . T . T . . . . . . . . . . . . . . . . . .    
548	. G G G G G G G G G G G G G G G G . . . G . G . . . . . . . . . . . . . . . . . .    
549	. G G G G G G G G G G G G G G G G . . . G . G . . . . . . . . . . . . . . . . . .    
550	. G G G G G G G G G G G G G G G G . . . G . G . . . . . . . . . . . . . . . . . .    
551	. G G G G G G G G G G G G G G G G . . . G . G . . . . . . . . . . . . . . . . . .    
552	. T T T T T T T T T T T T T T T T . . . T . T . . . . . . . . . . . . . . . . . .    
553	. A A A A A A A A A A A A A A A A . . . A . A . . . . . . . . . . . . . . . . . .    
554	. C C C C C C C C C C C C C C C C . . . C . C . . . . . . . . . . . . . . . . . .    
555	. C A A A A A A A A A A A A A A A . . . A . A . . . . . . . . . . . . . . . . . .    
556	. . C C C C C C C C C C C C C C C . . . C . C . . . . . . . . . . . . . . . . . .    
557	. . T T T T T T T T T T T T T T T . . . T . T . . . . . . . . . . . . . . . . . .    
558	. . C C C C C C C C C C C C C C C . . . C . C . . . . . . . . . . . . . . . . . .    
559	. . T T T T T T T T T T T T T T T . . . T . T . . . . . . . . . . . . . . . . . .    
560	. . G G G G G G G G G G G G G G G . . . G . G . . . . . . . . . . . . . . . . . .    
561	. . G G G G G G G G G G G G G G G . . . G . G . . . . . . . . . . . . . . . . . .    
562	. . C C C C C C C C C C C C C C C . . . C . C . . . . . . . . . . . . . . . . . .    
563	. . C C C C C C C C C C C C C C C . . . C . C . . . . . . . . . . . . . . . . . .    
564	. . A A A A A A A A A A A A A A A . . . A . A . . . . . . . . . . . . . . . . . .    
565	. . C C C C C C C C C C C C C C C . . . C . C . . . . . . . . . . . . . . . . . .    
566	. . A A A A A A A A A A A A A A A . . . A . A . . . . . . . . . . . . . . . . . .    
567	. . G G G G G G G G G G G G G G G . . . G . G . . . . . . . . . . . . . . . . . .    
568	. . A A A A A A A A A A A A A A A . . . A . A . . . . . . . . . . . . . . . . . .    
569	. . A A A A A A A A A A A A A A A . . . A . A . . . . . . . . . . . . . . . . . .    
570	. . G G G G G G G G G G G G G G G . . . G . G . . . . . . . . . . . . . . . . . .    
571	. . A A A A A A A A A A A A A A A . . . A . A . . . . . . . . . . . . . . . . . .    
572	. . G G G G G G G G G G G G G G G . . . G . G . . . . . . . . . . . . . . . . . .    
573	. . G G G G G G G G G G G G G G G . . . G . G . . . . . . . . . . . . . . . . . .    
574	. . C C C C C C C C C C C C C C C . . . C . C . . . . . . . . . . . . . . . . . .    
575	. . G G G G G G G G G G G G G G G . . . G . G . . . . . . . . . . . . . . . . . .    
576	. . G G G G G G G G G G G G G G G . . . G . G . . . . . . . . . . . . . . . . . .    
577	. . A A A A A A A A A A A A A A A . . . A . A . . . . . . . . . . . . . . . . . .    
578	. . G G G G G G G G G G G G G G G . . . G . G . . . . . . . . . . . . . . . . . .    
579	. . C C C C C C C C C C C C C C C . . . C . C . . . . . . . . . . . . . . . . . .    
580	. . G G G G G G G G G G G G G G G . . . G . G . . . . . . . . . . . . . . . . . .    
581	. . A A A A A A A A A A A A A A A . . . A . A . . . . . . . . . . . . . . . . . .    
582	. . A A A A A A A A A A A A A A A . . . A . A . . . . . . . . . . . . . . . . . .    
583	. . T T T T T T T T T T T T T T T . . . T . T . . . . . . . . . . . . . . . . . .    
584	. . C C C C T C C T C C T T C T C . . . C . T . . . . . . . . . . . . . . . . . .  6	 transition  
585	. . G G G G G G G G G G G G G G G . . . G . G . . . . . . . . . . . . . . . . . .    
586	. . G G G G G G G G G G G G G G G . . . G . G . . . G . . . . . . . . . . . . . .    
587	. . A A A A A A A A A A A A A A A . . . A . A . . . A . . . . . . . . . . . . . .    
588	. . G G G G G G G G G G G G G G G . . . G . G . . . G . G . . . . . . . . . . . .    
589	. . T T T T T T T T T T T T T T T . . . T . T . . . T . T . . . . . . . . . . . .    
590	. . G G G G G G G G G G G G G G G . . . G . G . . . G . G . . . . . . . . . . . .    
591	. . G G G G G G G G G G G G G G G . . . G . G . . . G . G . . . . . . . . . . . .    
592	. . A A A A A A A A A A A A A A A . . . A . A . . . A . A . . . . A . . . . . . .    
593	. . T T T T T T T T T T T T T T T . . . T . T . . . T . T . . . . T . . . . . . .    
594	. . C C C C C C C C C C C C C C C . . . C . C . . . C . C . . . . C . . . . . . .    
595	. . A A A A A A A A A A A A A A A . . . A . A . . . A . A . . . . A . . . . . . .    
596	. . C C C C C C C C C C C C C C C . . . C . C . . . C . C . . . . C . . . . . . .    
597	. . G G G G G G G G G G G G G G G . . . G . G . . . G . G . . . . G . . . . . . .    
598	. . T T T T T T T T T T T T T T T . . . T . T . . . T . T . . . . T . . . . . . .    
599	. . G G G G G G G G G G G G G G G . . . G . G . . . G . G . . . . G . . . . . . .    
600	. . T G G G G G G G G G G G G G G . . . G . G . . . G . G . . . . G . . . . . . .    
601	. . C C C C C C C C C C C C C C C . . . C . C . . . C . C . . . . C . . . . . . .    
602	. . T T T T T T T T T T T T T T T . . . T . T . . . T . T . . . . T . . . . . . .    
603	. . C C C C C C C C C C C C C C C . . . C . C . . . C . C . . . . C . . . . . . .    
604	. . G G G G G G G G G G G G G G G . . . G . G . . . G . G . . . . G . . . . . . .    
605	. . C C C C C C C C C C C C C C C . . . C . C . . . C . C . . . . C . . . . . . C    
606	. . A A A A A A A A A A A A A A A . . . A . A . . . A . A . . . . A . . . . . . A    
607	. . T T T T T T T T T T T T T T T . . . T . T . . . T . T . . . . T . . . . . . T    
608	. . G G G G G G G G G G G G G G G . . . G . G . . . G . G . . . G G . . . . . . G    
609	. . A A A A A A A A A A A A A A A . . . A . A . . . A . A . . . A A . . . . . . A    
610	. . C C C C C C C C C C C C C C C . . . C . C . . . C . C . . . C C . . . . . . C    
611	. . A A A A A A A A A A A A A A A . . . A . A . . . A . A . . . A A . . . . . . A    
612	. . G G G G G G G G G G G G G G G . . . G . G . . . G . G . . . G G . . . . . . G    
613	. . C C C C C C C C C C C C C C C . . . C . C . . . C . C . . . C C . . . . . . C    
614	. . C C C C C C C C C C C C C C C . . . C . C . . . C . C . . . C C . . . . . . C    
615	. . A A A A A A A A A A A A A A A . . . A . A . . . A . A . . . A A . . . . . . A    
616	. . C C C C C C C C C C C C C C C . . . C . C . . . C . C . . . C C . . . . . . C    
617	. . A A A A A A A A A A A A A A A . . . A . A . . . A . A . . . A A . . . . . . A    
618	. . G G G G G G G G G G G G G G G . . . G . G . . . G . G . . . G G . . . . . . G    
619	. . G G G G G G G G G G G G G G G . . . G . G . . . G . G . . . G G . . . . . . G    
620	. . C C C C C C C C C C C C C C C . . . C . C . . . C . C . . . C C . . . . . . C    
621	. . A A A A A A A A A A A A A A A . . . A . A . . . A . A . . . A A . . . . . . A    
622	. . C C C C C C C C C C C C C C C . . . C . C . . . C . C . . . C C . . . . . . C    
623	. . G G G G G G G G G G G G G G G . . . G . G . . . G . G . . . G G . . . . . . G    
624	. . G G G G G G G G G G G G G G G . . . G . G . . . G . G . . . G G . . . . . . G    
625	. . G G G G G G G G G G G G G G G . . . G . G . . . G . G . . . G G . . . . . . G    
626	. . G G G G G G G G G G G G G G G . . . G . G . . . G . G . . . G G . . . . . . G    
627	. . G G G G G G G G G G G G G G G . . . G . G . . . G . G . . . G G . . . . . . G    
628	. . A A A A A A A A A A A A A A A . . . A . A . . . A . A . . A A A . . . . . . A    
629	. . G G G G G G G G G G G G G G G . . . G . G . . . G . G . . G G G . . . . . . G    
630	. . A A A A A A A A A A A A A A A . . . A . A . . . A . A . . A A A . . . . . . A    
631	. . A A A A A A A A A A A A A A A . . . A . A . . . A . A . . A A A . . . . . . A    
632	. . T T T T T T T T T T T T T T T . . . T . T . . . T . T . . T T T . . . . . . T    
633	. . T T T T T T T T T T T T T T T . . . T . T . . . T . T . . T T T . . . . . . T    
634	. . C C C C C C C C C C C C C C C . . . C . C . . . C . C . . C C C . . . . . . C    
635	. . A A A A A A A A A A A A A A A . . . A . A . . . A . A . . A A A . . . . . . A    
636	. . A A A A A A A A A A A A A A A . . . A . A . . . A . A . . A A A . . . . . A A    
637	. . C C C C C C C C C C C C C C C . . . C . C . . . C . C . . C C C . . . . . C C    
638	. . A A A A A A A A A A A A A A A . . . A . A . . . A . A . . A A A . . . . . A A    
639	. . G G G G G G G G G G G G G G G . . . G . G . . . G . G . . G G G . . . . . G G    
640	. . T T T T T T T T T T T T T T T . . . T . T . . . T . T . . T T T . . . . . T T    
641	. . G G G G G G G G G G G G G G G . . . G . G . . . G . G . . G G G . . . . . G G    
642	. . . G G G G G G G G G G G G G G . . . G . G . . . G . G . . G G G . . . . . G G    
643	. . . C C C C C C C C C C C C C C . . . C . C . . . C . C . . C C C . . . . . C C    
644	. . . C C C C C C C C C C C C C C . . . C . C . . . C . C . . C C C . . . . . C C    
645	. . . G G G G G G G G G G G G G G . . . G . G . . . G . G . . G G G . . . . . G G    
646	. . . A A A A A A A A A A A A A A . . . A . A . . . A . A . . A A A . . . . . A A    
647	. . . A A A A A A A A A A A A A A . . . A . A . . . A . A . . A A A . . . . . A A    
648	. . . C C C C C C C C C C C C C C . . . C . C . . . C . C . . C C C . . . . . C C    
649	. . . A A A A A A A A A A A A A A . . . A . A . . . A . A . . A A A . . . . . A A    
650	. . . T T T T T T T T T T T T T T . . . T . T . . . T . T . . T T T . . . . . T T    
651	. . . C C C C C C C C C C C C C C . . . C . C . . . C . C . . C C C . . . . . C C    
652	. . . T T T T T T T T T T T T T T . . . T . T . . . T . T . . T T T . . . . . T T    
653	. . . C C C C C C C C C C C C C C . . . C . C . C . C . C . . C C C . . . . . C C    
654	. . . A A A A A A A A A A A A A A . . . A . A . A . A . A . . A A A . . . . . A A    
655	. . . T T T T T T T T T T T T T T . . . T . T . T . T . T . . T T T . . . . . T T    
656	. . . A A A A A A A A A A A A A A . . . A . A . A . A . A . . A A A . . . . . A A    
657	. . . G G G G G G G G G G G G G G . . . G . G . G . G . G . . G G G . . . . . G G    
658	. . . C C C C C C C C C C C C C C . . . C . C . C . C . C . . C C C . . . . . C C    
659	. . . A A A A A A A A A A A A A A . . . A . A . A . A . A . . A A A . . . . . A A    
660	. . . C C C C C C C C C C C C C C . . . C . C . C . C . C . . C C C . . . . . C C    
661	. . . A A A A A A A A A A A A A A . . . A . A . A . A . A . . A A A . . . . . A A    
662	. . . A A A A A A A A A A A A A A . . . A . A . A . A . A . . A A A . . . . . A A    
663	. . . A - - - - - - - - - - - - - . . . - . - . - . - . - . . - - - . . . . . - -    
664	. . . C C C C C C C C C C C C C C . . . C . C . C . C . C . . C C C . . . . . C C    
665	. . . A A A A A A A A A A A A A A . . . A . A . A . A . A . . A A A . . . . . A A    
666	. . . C C C C C C C C C C C C C C . . . C . C . C . C . C . . C C C . . . . . C C    
667	. . . A A A A A A A A A A A A A A . . . A . A . A . A . A . . A A A . . . . . A A    
668	. . . - - - - - - - - - - - - - - . . . - . - . - . A . - . . - - - . . . . . - -    
669	. . . G G G G G G G G G G G G G G . . . G . G . G . G . G . . G G G . . . . . G G    
670	. . . T T T T T T T T T T T T T T . . . T . T . T . T . T . . T T T . . T . . T T    
671	. . . G G G G G G G G G G G G G G . . . G . G . G . G . G . . G G G . . G . . G G    
672	. . . C C C C C C C C C C C C C C . . . C . C . C . C . C . . C C C . . C . . C C    
673	. . . A A A A A A A A A A A A A A . . . A . A . A . A . A . . A A A . . A . . A A    
674	. . . A A A A A A A A A A A A A A . . . A . A . A . A . A . . A A A . . A . A A A    
675	. . . T T T T T T T T T T T T T T . . . T . T . T . T . T . . T T T . . T . T T T    
676	. . . T T T T T T T T T T T T T T . . . T . T . T . T T T . . T T T . . T . T T T    
677	. . . A A A A A A A A A A A A A A . . . A . A . A . A A A . . A A A . . A . A A A    
678	. . . A A A A A A A A A A A A A A . . . A . A . A . A A A . . A A A . . A . A A A    
679	. . . G G G G G G G G G G G G G G . . . G . G . G . G G G . . G G G . . G . G G G    
680	. . . A A A A A A A A A A A A A A . . . A . A . A . A A A . . A A A . . A . A A A    
681	. . . T T T T T T T T T T T T T T . . . T . T . T . T T T . . T T T . . T . T T T    
682	. . . G G G G G G G G G G G G G G . . . G . G . G . G G G . . G G G . . G . G G G    
683	. . . C C C C C C C C C C C C C C . . . C . C . C . C C C . . C C C . . C . C C C    
684	. . . T T T T T T T T T T T T T T . . . T . T . T . T T T . . T T T . . T . T T T    
685	. . . C C C C C C C C C C C C C C . . . C . C . C . C C C . . C C C . . C . C C C    
686	. . . C C C C C C C C C C C C C C . . . C . C . C . C C C . . C C C . . C . C C C    
687	. . . A A A A A A A A A A A A A A . . . A . A . A . A A A . . A A A . . A . A A A    
688	. . . C C C C C C C C C C C C C C . . . C . C . C . C C C . . C C C . . C . C C C    
689	. . . A A A A A A A A A A A A A A . . . A . A . A . A A A . . A A A . A A . A A A    
690	. . . G G G G G G G G G G G G G G . . . G . G . G . G G G . . G G G . G G . G G G    
691	. . . C C C C C C C C C C C C C C . . . C . C . C . C C C . . C C C . C C . C C C    
692	. . . A A A A A A A A A A A A A A . . . A . A . A . A A A . . A A A . A A . A A A    
693	. . . G G G G G G G G G G G G G G . . . G . G . G . G G G . . G G G . G G . G G G    
694	. . . G G G G G G G G G G G G G G . . . G . G . G . G G G . . G G G . G G . G G G    
695	. . . G G G G G G G G G G G G G G . . . G . G . G . G G G . . G G G . G G . G G G    
696	. . . T T T T T T T T T T T T T T . . . T . T . T . T T T . . T T T . T T . T T T    
697	. . . G G G G G G G G G G G G G G . . . G . G . G . G G G G . G G G . G G . G G G    
698	. . . A A A A A A A A A A A A A A . . . A . A . A . A A A A . A A A . A A . A A A    
699	. . . A A A A A A A A A A A A A A . . . A . A . A . A A A A . A A A . A A . A A A    
700	. . . A A A A A A A A A A A A A A . . . A . A . A . A A A A . A A A . A A . A A A    
701	. . . G G G G G G G G G G G G G G . . . G . G . G . G G G G . G G G . G G . G G G    
702	. . . T T T T T T T T T T T T T T . . . T . T . T . T T T T . T T T . T T . T T T    
703	. . . C C C C C C C C C C C C C C . . . C . C . C . C C C C . C C C . C C . C C C    
704	. . . A A A A A A A A A A A A A A . . . A . A . A . A A A A . A A A . A A . A A A    
705	. . . T T T T T T T T T T T T T T . . . T . T . T . T T T T . T T T . T T . T T T    
706	. . . . C C C C C C C C C C C C C . . . C . C . C . C C C C . C C C . C C . C C C    
707	. . . . T T T T T T T T T T T T T . . . T . T . T . T T T T . T T T . T T . T T T    
708	. . . . T T T T T T T T T T T T T . . . T . T . T . T T T T . T T T . T T . T T T    
709	. . . . G G G G G G G G G G G G G . . . G . G . G . G G G G . G G G . G G . G G G    
710	. . . . G G G G G G G G G G G G G . . . G . G . G . G G G G . G G G . G G . G G G    
711	. . . . A A A A A A A A A A A A A . . . A . A . A . A A A A . A A A . A A . A A A    
712	. . . . A A A A A A A A A A A A A . . . A . A . A . A A A A . A A A . A A . A A A    
713	. . . . T T T T T T T T T T T T T . . . T . T . T . T T T T . T T T . T T . T T T    
714	. . . . A A A A A A A A A A A A A . . . A . A . A . A A A A . A A A . A A . A A A    
715	. . . . T T T T T T T T T T T T T . . . T . T . T . T T T T . T T T . T T . T T T    
716	. . . . G G G G G G G G G G G G G . . . G . G . G . G G G G . G G G . G G . G G G    
717	. . . . T T T T T T T T T T T T T . . . T T T . T . T T T T T T T T . T T . T T T    
718	. . . . C C C C C C C C C C C C C . . . C C C . C . C C C C C C C C . C C . C C C    
719	. . . . A A A A A A A A A A A A A . . . A A A . A . A A A A A A A A . A A . A A A    
720	. . . . A A A A A A A A A A A A A . . . A A A . A . A A A A A A A A . A A . A A A    
721	. . . . A A A A A A A A A A A A A . . . A A A . A . A A A A A A A A . A A . A A A    
722	. . . . G G G G G G G G G G G G G . . . G G G . G . G G G G G G G G . G G . G G G    
723	. . . . C C C C C C C C C C C C C . . . C C C . C . C C C C C C C C . C C . C C C    
724	. . . . A A A A A A A A A A A A A . . . A A A . A . A A A A A A A A . A A . A A A    
725	. . . . G G G G G G G G G G G G G . . . G G G . G . G G G G G G G G . G G . G G G    
726	. . . . T T T T T T T T T T T T T . . . T T T . T . T T T T T T T T . T T . T T T    
727	. . . . G G G G G G G G G G G G G . . . G G G . G . G G G G G G G G . G G . G G G    
728	. . . . G G G G G G G G G G G G G . . . G G G . G . G G G G G G G G . G G . G G G    
729	. . . . A A A A A A A A A A A A A . . . A A A . A . A A A A A A A A . A A . A A A    
730	. . . . A A A A A A A A A A A A A . . . A A A . A . A A A A A A A A . A A . A A A    
731	. . . . G G G G G G G G G G G G G . . . G G G . G . G G G G G G G G . G G G G G G    
732	. . . . C C C C C C C C C C C C C . . . C C C . C . C C C C C C C C . C C C C C C    
733	. . . . A A A A A A A A A A A A A . . . A A A . A . A A A A A A A A . A A A A A A    
734	. . . . G G G G G G G G G G G G G . . . G G G . G . G G G G G G G G . G G G G G G    
735	. . . . G G G G G G G G G G G G G . . . G G G . G . G G G G G G G G . G G G G G G    
736	. . . . A A . A A A A A A A A A A . . . A A A . A . A A A A A A A A . A A A A A A    
737	. . . . G G . G G G G G G G G G G . . . G G G G G . G G G G G G G G . G G G G G G    
738	. . . . A A . A A A A A A A A A A . . . A A A A A . A A A A A A A A . A A A A A A    
739	. . . . G G . G G G G G G G G G G . . . G G G G G . G G G G G G G G . G G G G G G    
740	. . . . G G . G G G G G G G G G G . . . G G G G G . G G G G G G G G . G G G G G G    
741	. . . . T T . T T T T T T T T T T . . . T T T T T . T T T T T T T T . T T T T T T    
742	. . . . G G . G G G G G G G G G G . . . G G G G G . G G G G G G G G . G G G G G G    
743	. . . . C C . C C C C C C C C C C . . . C C C C C . C C C C C C C C . C C C C C C    
744	. . . . C C . C C C C C C C C C C . . . C C C C C . C C C C C C C C . C C C C C C    
745	. . . . A A . A A A A A A A A A A . . . A A A A A . A A A A A A A A . A A A A A A    
746	. . . . T T . T T T T T T T T T T . . . T T T T T . T T T T T T T T . T T T T T T    
747	. . . . T T . T T T T T T T T T T . . . T T T T T . T T T T T T T T . T T T T T T    
748	. . . . T T . T T T T T T T T T T . . . T T T T T . T T T T T T T T . T T T T T T    
749	. . . . A A . A A A A A A A A A A . . . A A A A A . A A A A A A A A . A A A A A A    
750	. . . . A A . A A A A A A A A A A . . . A A A A A . A A A A A A A A . A A A A A A    
751	. . . . C C . C C C C C C C C C C . . . C C C C C . C C C C C C C C . C C C C C C    
752	. . . . C C . C C C C C C C C C C . . . C C C C C . C C C C C C C C . C C C C C C    
753	. . . . A A . A A A A A A A A A A . . . A A A A A . A A A A A A A A . A A A A A A    
754	. . . . C C . C C C C C C C C C C . . . C C C C C . C C C C C C C C . C C C C C C    
755	. . . . G G . G G G G G G G G G G . . . G G G G G . G G G G G G G G . G G G G G G    
756	. . . . A A . A A A A A A A A A A . . . A A A A A . A A A A A A A A . A A A A A A    
757	. . . . G G . G G G G G G G G G G . . . G G G G G . G G G G G G G G . G G G G G G    
758	. . . . A . . A A A A A A A A A A . . . A A A A A . A A A A A A A A . A A A A A A    
759	. . . . T . . T T T T T T T T T T . . . T T T T T . T T T T T T T T . T T T T T T    
760	. . . . C . . C C C C C C C C C C . . . C C C C C . C C C C C C C C . C C C C C C    
761	. . . . C . . C C C C C C C C C C . . . C C C C C . C C C C C C C C . C C C C C C    
762	. . . . T . . T T T T T T T T T T . . . T T T T T . T T T T T T T T . T T T T T T    
763	. . . . C . . C C C C C C C C C C . . . C C C C C . C C C C C C C C . C C C C C C    
764	. . . . A . . A A A A A A A A A A . . . A A A A A . A A A A A A A A . A A A A A A    
765	. . . . A . . G G G G G G G G G G . . . G G G G G . G G G G G G G G . G G G G G G    
766	. . . . . . . A A A A A A A A A A . . . A A A A A . A A A A A A A A . A A A A A A    
767	. . . . . . . G G G G G G G G G G . . . G G G G G . G G G G G G G G . G G G G G G    
768	. . . . . . . A A A A A A A A A A . . . A A A A A . A A A A A A A A . A A A A A A    
769	. . . . . . . A A A A A A A A A A . . . A A A A A . A A A A A A A A . A A A A A A    
770	. . . . . . . G G G G G G G G G G . . . G G G G G . G G G G G G G G . G G G G G G    
771	. . . . . . . C C C C C C C C C C . . . C C C C C . C C C C C C C C . C C C C C C    
772	. . . . . . . C C C C C C C C C C . . . C C C C C . C C C C C C C C . C C C C C C    
773	. . . . . . . A A A A A A A A A A . . . A A A A A . A A A A A A A A . A A A A A A    
774	. . . . . . . A A A A A A A A A A . . . A A A A A . A A A A A A A A . A A A A A A    
775	. . . . . . . T T T T T T T T T T . . . T T T T T . T T T T T T T T . T T T T T T    
776	. . . . . . . G G G G G G G G G G . . . G G G G G . G G G G G G G G . G G G G G G    
777	. . . . . . . C C C C C C C C C C . . . C C C C C . C C C C C C C C . C C C C C C    
778	. . . . . . . C C C C C C C C C C . . . C C C C C . C C C C C C C C . C C C C C C    
779	. . . . . . . C C C C C C C C C C . . . C C C C C . C C C C C C C C . C C C C C C    
780	. . . . . . . T T T T T T T T T T . . . T T T T T T T T T T T T T T . T T T T T T    
781	. . . . . . . G G G G G G G G G G . . . G G G G G G G G G G G G G G . G G G G G G    
782	. . . . . . . T T T T T T T T T T . . . T T T T T T T T T T T T T T . T T T T T T    
783	. . . . . . . G G G G G G G G G G . . G G G G G G G G G G G G G G G . G G G G G G    
784	. . . . . . . C C C C C C C C C C . . C C C C C C C C C C C C C C C . C C C C C C    
785	. . . . . . . C C C C C C C C C C . . C C C C C C C C C C C C C C C . C C C C C C    
786	. . . . . . . A A A A A A A A A A . . A A A A A A A A A A A A A A A . A A A A A A    
787	. . . . . . . T T T T T T T T T T . . T T T T T T T T T T T T T T T . T T T T T T    
788	. . . . . . . A A A A A A A A A A . . A A A A A A A A A A A A A A A . A A A A A A    
789	. . . . . . . G A G G G G G G G G . . G G G G G G G G G G G G G G G . G G G G G G    
790	. . . . . . . A . A A A A A A A A . . A A A A A A A A A A A A A A A . A A A A A A    
791	. . . . . . . C . C C C C C C C C . . C C C C C C C C C C C C C C C . C C C C C C    
792	. . . . . . . T . T T T T T T T T . . T T T T T T T T T T T T T T T . T T T T T T    
793	. . . . . . . G . G G G G G G G G . . G G G G G G G G G G G G G G G . G G G G G G    
794	. . . . . . . C . C C C C C C C C . . C C C C C C C C C C C C C C C . C C C C C C    
795	. . . . . . . C . C C C C C C C C . . C C C C C C C C C C C C C C C . C C C C C C    
796	. . . . . . . T . T T T T T T T T . . T T T T T T T T T T T T T T T . T T T T T T    
797	. . . . . . . G . G G G G G G G G . . G G G G G G G G G G G G G G G . G G G G G G    
798	. . . . . . . T . T T T T T T T T . . T T T T T T T T T T T T T T T . T T T T T T    
799	. . . . . . . T . T T T T T T T T . . T T T T T T T T T T T T T T T . T T T T T T    
800	. . . . . . . C . C C C C C C C C . . C C C C C C C C C C C C C C C . C C C C C C    
801	. . . . . . . T . T T T T T T T T . . T T T T T T T T T T T T T T T T T T T T T T    
802	. . . . . . . C . C C C C C C C C . . C C C C C C C C C C C C C C C C C C C C C C    
803	. . . . . . . A . A A A A A A A A . . A A A A A A A A A A A A A A A A A A A A A A    
804	. . . . . . . G . G G G G G G G G . . G G G G G G G G G G G G G G G G G G G G G G    
805	. . . . . . . C . C C C C C C C C . . C C C C C C C C C C C C C C C C C C C C C C    
806	. . . . . . . A . A A A A A A A A . . A A A A A A A A A A A A A A A A A A A A A A    
807	. . . . . . . C . C C C C C C C C . . C C C C C C C C C C C C C C C C C C C C C C    
808	. . . . . . . C . C C C C C C C C . . C C C C C C C C C C C C C C C C C C C C C C    
809	. . . . . . . A . A A A A A A A A . . A A A A A A A A A A A A A A A A A A A A A A    
810	. . . . . . . T . T T T T T T T T . . T T T T T T T T T T T T T T T T T T T T T T    
811	. . . . . . . C . C C C C C C C C . . C C C C C C C C C C C C C C C C C C C C C C    
812	. . . . . . . A . A A A A A A A A . . A A A A A A A A A A A A A A A A A A A A A A    
813	. . . . . . . A . A A A A A A A A . . A A A A A A A A A A A A A A A A A A A A A A    
814	. . . . . . . A . A A A A A A A A . . A A A A A A A A A A A A A A A A A A A A A A    
815	. . . . . . . T . T T T T T T T T . . T T T T T T T T T T T T T T T T T T T T T T    
816	. . . . . . . T . T T T T T T T T . . T T T T T T T T T T T T T T T T T T T T T T    
817	. . . . . . . C . C T C C C C C C . . C C C C C C C C C T C C C C C C C C C C C C    
818	. . . . . . . A . A A A A A A A A . . A A A A A A A A A A A A A A A A A A A A A A    
819	. . . . . . . A . A A A A A A A A . . A A A A A A A A A A A A A A A A A A A A A A    
820	. . . . . . . G . G G G G G G G G . . G G G G G G G G G G G G G G G G G G G G G G    
821	. . . . . . . A . A A A A A A A A . . A A A A A A A A A A A A A A A A A A A A A A    
822	. . . . . . . C . C C C C C C C C . . C C C C C C C C C C C C C C C C C C C C C C    
823	. . . . . . . T . T T T T T T T T . . T T T T T T T T T T T T T T T T T T T T T T    
824	. . . . . . . G . G G G G G G G G . . G G G G G G G G G G G G G G G G G G G G G G    
825	. . . . . . . A . A A A A A A A A . . A A A A A A A A A A A A A A A A A A A A A A    
826	. . . . . . . C . C C C C C C C C . . C C C C C C C C C C C C C C C C C C C C C C    
827	. . . . . . . T . T T T T T T T T . . T T T T T T T T T T T T T T T T T T T T C T    
828	. . . . . . . . . T T T T T T T T . . T T T T T T T T T T T T T T T T T T T T T T    
829	. . . . . . . . . C C C C C C C C . . C C C C C C C C C C C C C C C C C C C C C C    
830	. . . . . . . . . T T T T T T T T . . T T T T T T T T T T T T T T T T T T T T T T    
831	. . . . . . . . . A A A A A A A A . . A A A A A A A A A A A A A A A A A A A A A A    
832	. . . . . . . . . T T T T T T T T . . T T T T T T T T T T T T T T T T T T T T T T    
833	. . . . . . . . . G G G G G G G G . . G G G G G G G G G G G G G G G G G G G G G G    
834	. . . . . . . . . . A A A A A A A . . A A A A A A A A A A A A A A A A A A A A A A    
835	. . . . . . . . . . T T T T T T T . . T T T T T T T T T T T T T T T T T T T T T T    
836	. . . . . . . . . . C C C C C C C . . C C C C C C C C C C C C C C C C C C C C C C    
837	. . . . . . . . . . A A A A A A A . . A A A A A A A A A A A A A A A A A A A A A A    
838	. . . . . . . . . . A A A A A A A . . A A A A A A A A A A A A A A A A A A A A A A    
839	. . . . . . . . . . T T T T T T T . . T T T T T T T T T T T T T T T T T T T T T T    
840	. . . . . . . . . . G G G G G G G . . G G G G G G G G G G G G G G G G G G G G G G    
841	. . . . . . . . . . C C C C C C C . . C C C C C C C C C C C C C C C C C C C C C C    
842	. . . . . . . . . . A A A A A A A . . A A A A A A A A A A A A A A A A A A A A A A    
843	. . . . . . . . . . A A A A A A A . . A A A A A A A A A A A A A A A A A A A A A A    
844	. . . . . . . . . . T T T T T T T . . T T T T T T T T T T T T T T T T T T T T T T    
845	. . . . . . . . . . G G G G G G G . . G G G G G G G G G G G G G G G G G G G G G G    
846	. . . . . . . . . . A A A A A A A . . A A A A A A A A A A A A A A A A A A A A A A    
847	. . . . . . . . . . . C C C C C C . . C C C C C C C C C C C C C C C C C C C C C C    
848	. . . . . . . . . . . G G G G G G . . G G G G G G G G G G G G G G G G G G G G G G    
849	. . . . . . . . . . . T T T T T T . . T T T T T T T T T T T T T T T T T T T T T T    
850	. . . . . . . . . . . G G G G G G . . G G G G G G G G G G G G G G G G G G G G G G    
851	. . . . . . . . . . . G G G G G G . . G G G G G G G G G G G G G G G G G G G G G G    
852	. . . . . . . . . . . G G G G G G . . G G G G G G G G G G G G G G G G G G G G G G    
853	. . . . . . . . . . . C C C C C C . . C C C C C C C C C C C C C C C C C C C C C C    
854	. . . . . . . . . . . C C C C C C . . C C C C C C C C C C C C C C C C C C C C C C    
855	. . . . . . . . . . . T T T T T T . . T T T T T T T T T T T T T T T T T T T T T T    
856	. . . . . . . . . . . C C C C C C . . C C C C C C C C C C C C C C C C C C C C C C    
857	. . . . . . . . . . . A A A A A A . . A A A A A A A A A A A A A A A A A A A A A A    
858	. . . . . . . . . . . T T T T T T . . T T T T T T T T T T T T T T T T T T T T T T    
859	. . . . . . . . . . . G . G G G G . . G G G G G G G G G G G G G G G G G G G G G G    
860	. . . . . . . . . . . G . G G G G . . G G G G G G G G G G G G G G G G G G G G G G    
861	. . . . . . . . . . . C . C C C C . . C C C C C C C C C C C C C C C C C C C C C C    
862	. . . . . . . . . . . T . T T T T . . T T T T T T T T T T T T T T T T T T T T T T    
863	. . . . . . . . . . . T . T T T T . . T T T T T T T T T T T T T T T T T T T T T T    
864	. . . . . . . . . . . A . A A A A . . A A A A A A A A A A A A A A A A A A A A A A    
865	. . . . . . . . . . . C . C C C C . . C C C C C C C C C C C C C C C C C C C C C C    
866	. . . . . . . . . . . C . C C C C . . C C C C C C C C C C C C C C C C C C C C C C    
867	. . . . . . . . . . . T . T T T T . . T T T T T T T T T T T T T T T T T T T T T T    
868	. . . . . . . . . . . A . A A A A . . A A A A A A A A A A A A A A A A A A A A A A    
869	. . . . . . . . . . . G . G G G G . . G G G G G G G G G G G G G G G G G G G G G G    
870	. . . . . . . . . . . G . G G G G . . G G G G G G G G G G G G G G G G G G G G G G    
871	. . . . . . . . . . . T . T T T T . . T T T T T T T T T T T T T T T T T T T T T T    
872	. . . . . . . . . . . A . A A A A . . A A A A A A A A A A A A A A A A A A A A A A    
873	. . . . . . . . . . . C . C C C C . . C C C C C C C C C C C C C C C C C C C C C C    
874	. . . . . . . . . . . C . C C C C . . C C C C C C C C C C C C C C C C C C C C C C    
875	. . . . . . . . . . . A . A A A A . . A A A A A A A A A A A A A A A A A A A A G A    
876	. . . . . . . . . . . T . T T T T . . T T T T T T T T T T T T T T T T T T T T T T    
877	. . . . . . . . . . . C . C C C C . . C C C C C C C C C C C C C C C C C C C C C C    
878	. . . . . . . . . . . A . A A A A . . A A A A A A A A A A A A A A A A A A A A A A    
879	. . . . . . . . . . . C . C C C C . . C C C C C C C C C C C C C C C C C C C C C C    
880	. . . . . . . . . . . T . T T T T . . T T T T T T T T T T T T T T T T T T T T T T    
881	. . . . . . . . . . . A . A A A A . . A A A A A A A A A A A A A A A A A A A A A A    
882	. . . . . . . . . . . . . A A A A . . A A A A A A A A A A A A A A A A A A A A A A    
883	. . . . . . . . . . . . . G G G G . . G G G G G G G G G G G G G G G G G G G G G G    
884	. . . . . . . . . . . . . A A A A . . A A A A A A A A A A A A A A A A A A A A A A    
885	. . . . . . . . . . . . . C C C C . . C C C C C C C C C C C C C C C C C C C C C C    
886	. . . . . . . . . . . . . C C C C . . C C C C C C C C C C C C C C C C C C C C C C    
887	. . . . . . . . . . . . . T T T T . . T T T T T T T T T T T T T T T T T T T T T T    
888	. . . . . . . . . . . . . G G G G . . G G G G G G G G G G G G G G G G G G G G G G    
889	. . . . . . . . . . . . . C C C C . . C C C C C C C C C C C C C C C C C C C C C C    
890	. . . . . . . . . . . . . A A A A . . A A A A A A A A A A A A A A A A A A A A A A    
891	. . . . . . . . . . . . . A A A A . . A A A A A A A A A A A A A A A A A A A A A A    
892	. . . . . . . . . . . . . C C C C . . C C C C C C C C C C C C C C C C C C C C C C    
893	. . . . . . . . . . . . . A A A A . . A A A A A A A A A A A A A A A A A A A A A A    
894	. . . . . . . . . . . . . G G G G . . G G G G G G G G G G G G G G G G G G G G G G    
895	. . . . . . . . . . . . . C C C C . . C C C C C C C C C C C C C C C C C C C C C C    
896	. . . . . . . . . . . . . A A A A . . A A A A A A A A A A A A A A A A A A A A A A    
897	. . . . . . . . . . . . . . T T T . . T T T T T T T T T T T T T T T T T T T T T T    
898	. . . . . . . . . . . . . . G G G . . G G G G G G G G G G G G G G G G G G G G G G    
899	. . . . . . . . . . . . . . G A A . . A A A A A A A A A A A A G A A A A A A A A A    
900	. . . . . . . . . . . . . . A A A . . A A A A A A A A A A A A A A A A A A A A A A    
901	. . . . . . . . . . . . . . C C C . . C C C C C C C C C C C C C C C C C C C C C C    
902	. . . . . . . . . . . . . . C C C . . C C C C C C C C C C C C C C C C C C C C C C    
903	. . . . . . . . . . . . . . A A A . . A A A A A A A A A A A A A A A A A A A A A A    
904	. . . . . . . . . . . . . . G G G . . G G G G G G G G G G G G G G G G G G G G G G    
905	. . . . . . . . . . . . . . T T T . . T T T T T T T T T T T T T T T T T T T T T T    
906	. . . . . . . . . . . . . . T T T . . T T T T T T T T T T T T T T T T T T T T T T    
907	. . . . . . . . . . . . . . C C C . . C C C C C C C C C C C C C C C C C C C C C C    
908	. . . . . . . . . . . . . . A A A . . A A A A A A A A A A A A A A A A A A A A A A    
909	. . . . . . . . . . . . . . T T T . . T T T T T T T T T T T T T T T T T T T T T T    
910	. . . . . . . . . . . . . . C C C . . C C C C C C C C C C C C C C C C C C C C C C    
911	. . . . . . . . . . . . . . A A A . . A A A A A A A A A A A A A A A A A A A A A A    
912	. . . . . . . . . . . . . . A A A . . A A A A A A A A A A A A A A A A A A A A A A    
913	. . . . . . . . . . . . . . C C C . . C C C C C C C C C C C C C C C C C C C C C C    
914	. . . . . . . . . . . . . . A A A . . A A A A A A A A A A A A A A A A A A A A A A    
915	. . . . . . . . . . . . . . A A A . . A A A A A A A A A A A A A A A A A A A A A A    
916	. . . . . . . . . . . . . . G G G . . G G G G G G G G G G G G G G G G G G G G G G    
917	. . . . . . . . . . . . . . T T T . . T T T T T T T T T T T T T T T T T T T T T T    
918	. . . . . . . . . . . . . . T T T . . T T T T T T T T T T T T T T T T T T T T T T    
919	. . . . . . . . . . . . . . C C C . . C C C C C C C C C C C C C C C C C C C C C C    
920	. . . . . . . . . . . . . . A A A . . A A A A A A A A A A A A A A A A A A A A A A    
921	. . . . . . . . . . . . . . A A A . . A A A A A A A A A A A A A A A A A A A A A A    
922	. . . . . . . . . . . . . . C C C . . C C C C C C C C C C C C C C C C C C C C C C    
923	. . . . . . . . . . . . . . G G G . . G G G G G G G G G G G G G G G G G G G G G G    
924	. . . . . . . . . . . . . . T T T . . T T T T T T T T T T T T T T T T T T T T T T    
925	. . . . . . . . . . . . . . C C C . . C C C C C C C C C C C C C C C C C C C C C C    
926	. . . . . . . . . . . . . . T T T . . T T T T T T T T T T T T T T T T T T T T T T    
927	. . . . . . . . . . . . . . T T T . . T T T T T T T T T T T T T T T T T T T T T T    
928	. . . . . . . . . . . . . . G G G . . G G G G G G G G G G G G G G G G G G G G G G    
929	. . . . . . . . . . . . . . T T T . . T T T T T T T T T T T T T T T T T T T T T T    
930	. . . . . . . . . . . . . . A A A A . A A A A A A A A A A A A A A A A A A A A A A    
931	. . . . . . . . . . . . . . C C C C . C C C C C C C C C C C C C C C C C C C C C C    
932	. . . . . . . . . . . . . . G G G G G G G G G G G G G G G G G G G G G G G G G G G    
933	. . . . . . . . . . . . . . A . A A A A A A A A A A A A A A A A A A A A A A A A A    
934	. . . . . . . . . . . . . . T . T T T T T T T T T T T T T T T T T T T T T T T T T    
935	. . . . . . . . . . . . . . A . A A A A A A A A A A A A A A A A A A A A A A A A A    
936	. . . . . . . . . . . . . . G . G G G G G G G G G G G G G G G G G G G G G G G G G    
937	. . . . . . . . . . . . . . A . A A A A A A A A A A A A A A A A A A A A A A A A A    
938	. . . . . . . . . . . . . . C . C C C C C C C C C C C C C C C C C C C C C C C C C    
939	. . . . . . . . . . . . . . . . A A A A A A A A A A A A A A A A A A A A A A A A A    
940	. . . . . . . . . . . . . . . . G G G G G G G G G G G G G G G G G G G G G G G G G    
941	. . . . . . . . . . . . . . . . G G G G G G G G G G G G G G G G G G G G G G G G G    
942	. . . . . . . . . . . . . . . . G G G G G G G G G G G G G G G G G G G G G G G G G    
943	. . . . . . . . . . . . . . . . C C C C C C C C C C C C C C C C C C C C C C C C C    
944	. . . . . . . . . . . . . . . . A A A A A A A A A A A A A A A A A A A A A A A A A    
945	. . . . . . . . . . . . . . . . T T T T T T T T T T T T T T T T T T T T T T T T T    
946	. . . . . . . . . . . . . . . . C C C C C C C C C C C C C C C C C C C C C C C C C    
947	. . . . . . . . . . . . . . . . G G G G G G G G G G G G G G G G G G G G G G G G G    
948	. . . . . . . . . . . . . . . . G G G G G G G G G G G G G G G G G G G G G G G G G    
949	. . . . . . . . . . . . . . . . C C C C C C C C C C C C C C C C C C C C C C C C C    
950	. . . . . . . . . . . . . . . . C C C C C C C C C C C C C C C C C C C C C C C C C    
951	. . . . . . . . . . . . . . . . G G G G G G G G G G G G G G G G G G G G G G G G G    
952	. . . . . . . . . . . . . . . . G G G G G G G G G G G G G G G G G G G G G G G G G    
953	. . . . . . . . . . . . . . . . A A A A A A A A A A A A A A A A A A A A A A A A A    
954	. . . . . . . . . . . . . . . . G G G G G G G G G G G G G G G G G G G G G G G G G    
955	. . . . . . . . . . . . . . . . G G G G G G G G G G G G G G G G G G G G G G G G G    
956	. . . . . . . . . . . . . . . . A A A A A A A A A A A A A A A A A A A A A A A A A    
957	. . . . . . . . . . . . . . . . T T T T T T T T T T T T T T T T T T T T T T T T T    
958	. . . . . . . . . . . . . . . . G G G G G G G G G G G G G G G G G G G G G G G G G    
959	. . . . . . . . . . . . . . . . A A A A A A A A A A A A A A A A A A A A A A A A A    
960	. . . . . . . . . . . . . . . . G G G G G G G G G G G G G G G G G G G G G G G G G    
961	. . . . . . . . . . . . . . . . A A A A A A A A A A A A A A A A A A A A A A A A A    
962	. . . . . . . . . . . . . . . . G G G G G G G G G G G G G G G G G G G G G G G G G    
963	. . . . . . . . . . . . . . . . G G G G G G G G G G G G G G G G G G G G G G G G G    
964	. . . . . . . . . . . . . . . . A A A A A A A A A A A A A A A A A A A A A A A A A    
965	. . . . . . . . . . . . . . . . C C C C C C C C C C C C C C C C C C C C C C C C C    
966	. . . . . . . . . . . . . . . . T T T T T T T T T T T T T T T T T T T T T T T T T    
967	. . . . . . . . . . . . . . . . G G G G G G G G G G G G G G G G G G G G G G G G G    
968	. . . . . . . . . . . . . . . . T T T T T T T T T T T T T T T T T T T T T T T T T    
969	. . . . . . . . . . . . . . . . T T T T T T T T T T T T T T T T T T T T T T T T T    
970	. . . . . . . . . . . . . . . . C C C C C C C C C C C C C C C C C C C C C C C C C    
971	. . . . . . . . . . . . . . . . T T T T T T T T T T T T T T T T T T T T T T T T T    
972	. . . . . . . . . . . . . . . . T T T T T T T T T T T T T T T T T T T T T T T T T    
973	. . . . . . . . . . . . . . . . T T T T T T T T T T T T T T T T T T T T T T T T T    
974	. . . . . . . . . . . . . . . . . T T T T T T T T T T T T T T T T T T T T T T T T    
975	. . . . . . . . . . . . . . . . . G G G G G G G G G G G G G G G G G G G G G G G G    
976	. . . . . . . . . . . . . . . . . A A A A A A A A A A A A A A A A A A A A A A A A    
977	. . . . . . . . . . . . . . . . . G G G G G G G G G G G G G G G G G G G G G G G G    
978	. . . . . . . . . . . . . . . . . T T T T T T T T T T T T T T T T T T T T T T T T    
979	. . . . . . . . . . . . . . . . . G G G G G G G G G G G G G G G G G G G G G G G G    
980	. . . . . . . . . . . . . . . . . A A A A A A A A A A A A A A A A A A A A A A A A    
981	. . . . . . . . . . . . . . . . . C C C C C C C C C C C C C C C C C C C C C C C C    
982	. . . . . . . . . . . . . . . . . A A A A A A A A A A A A A A A A A A A A A A A A    
983	. . . . . . . . . . . . . . . . . C C C C C C C C C C C C C C C C C C C C C C C C    
984	. . . . . . . . . . . . . . . . . A A A A A A A A A A A A A A A A A A A A A A A A    
985	. . . . . . . . . . . . . . . . . G G G G G G G G G G G G G G G G G G G G G G G G    
986	. . . . . . . . . . . . . . . . . A A A A A A A A A A A A A A A A A A A A A A A A    
987	. . . . . . . . . . . . . . . . . A A A A A A A A A A A A A A A A A A A A A A A A    
988	. . . . . . . . . . . . . . . . . A A A A A A A A A A A A A A A A A A A A A A A A    
989	. . . . . . . . . . . . . . . . . G G G G G G G G G G G G G G G G G G G G G G G G    
990	. . . . . . . . . . . . . . . . . A A A A A A A A A A A A A A A A A A A A A A A A    
991	. . . . . . . . . . . . . . . . . G G G G G G G G G G G G G G G G G G G G G G G G    
992	. . . . . . . . . . . . . . . . . T T T T T T T T T T T T T T T T T T T T T T T T    
993	. . . . . . . . . . . . . . . . . G G G G G G G G G G G G G G G G G G G G G G G G    
994	. . . . . . . . . . . . . . . . . A A A A A A A A A A A A A A A A A A A A A A A A    
995	. . . . . . . . . . . . . . . . . A A A A A A A A A A A A A A A A A A A A A A A A    
996	. . . . . . . . . . . . . . . . . T T T T T T T T T T T T T T T T T T T T T T T T    
997	. . . . . . . . . . . . . . . . . G G G G G G G G G G G G G G G G G G G G G G G G    
998	. . . . . . . . . . . . . . . . . G G G G G G G G G G G G G G G G G G G G G G G G    
999	. . . . . . . . . . . . . . . . . A A A A A A A A A A A A A A A A A A A A A A A A    
1000	. . . . . . . . . . . . . . . . . G G G G G G G G G G G G G G G G G G G G G G G G    
1001	. . . . . . . . . . . . . . . . . G G G G G G G G G G G G G G G G G G G G G G G G    
1002	. . . . . . . . . . . . . . . . . A A A A A A A A A A A A A A A A A A A A A A A A    
1003	. . . . . . . . . . . . . . . . . A A A A A A A A A A A A A A A A A A A A A A A A    
1004	. . . . . . . . . . . . . . . . . G G G G G G G G G G G G G G G G G G G G G G G G    
1005	. . . . . . . . . . . . . . . . . A A A A A A A A A A A A A A A A A A A A A A A A    
1006	. . . . . . . . . . . . . . . . . G G G G G G G G G G G G G G G G G G G G G G G G    
1007	. . . . . . . . . . . . . . . . . A A A A A A A A A A A A A A A A A A A A A A A A    
1008	. . . . . . . . . . . . . . . . . T T T T T T T T T T T T T T T T T T T T T T T T    
1009	. . . . . . . . . . . . . . . . . G G G G G G G G G G G G G G G G G G G G G G G G    
1010	. . . . . . . . . . . . . . . . . G G G G G G G G G G G G G G G G G G G G G G G G    
1011	. . . . . . . . . . . . . . . . . A A A A A A A A A A A A A A A A A A A A A A A A    
1012	. . . . . . . . . . . . . . . . . G G G G G G G G G G G G G G G G G G G G G G G G    
1013	. . . . . . . . . . . . . . . . . A A A A A A A A A A A A A A A A A A A A A A A A    
1014	. . . . . . . . . . . . . . . . . G G G G G G G G G G G G G G G G G G G G G G G G    
1015	. . . . . . . . . . . . . . . . . G G G G G G G G G G G G G G G G G G G G G G G G    
1016	. . . . . . . . . . . . . . . . . T T T T T T T T T T T T T T T T T T T T T T T T    
1017	. . . . . . . . . . . . . . . . . C C C C C C C C C C C C C C C C C C C C C C C C    
1018	. . . . . . . . . . . . . . . . . A A A A A A A A A A A A A A A A A A A A A A A A    
1019	. . . . . . . . . . . . . . . . . C C C C C C C C C C C C C C C C C C C C C C C C    
1020	. . . . . . . . . . . . . . . . . A A A A A A A A A A A A A A A A A A A A A A A A    
1021	. . . . . . . . . . . . . . . . . A A A A A A A A A A A A A A A A A A A A A A A A    
1022	. . . . . . . . . . . . . . . . . G G G G G G G G G G G G G G G G G G G G G G G G    
1023	. . . . . . . . . . . . . . . . . C C C C C C C C C C C C C C C C C C C C C C C C    
1024	. . . . . . . . . . . . . . . . . A A A A A A A A A A A A A A A A A A A A A A A A    
1025	. . . . . . . . . . . . . . . . . C C C C C C C C C C C C C C C C C C C C C C C C    
1026	. . . . . . . . . . . . . . . . . T T T T T T T T T T T T T T T T T T T T T T T T    
1027	. . . . . . . . . . . . . . . . . G G G G G G G G G G G G G G G G G G G G G G G G    
1028	. . . . . . . . . . . . . . . . . C C C C C C C C C C C C C C C C C C C C C C C C    
1029	. . . . . . . . . . . . . . . . . T T T T T T T T T T T T T T T T T T T T T T T T    
1030	. . . . . . . . . . . . . . . . . T T T T T T T T T T T T T T T T T T T T T T T T    
1031	. . . . . . . . . . . . . . . . . T T T T T T T T T T T T T T T T T T T T T T T T    
1032	. . . . . . . . . . . . . . . . . T T T T T T T T T T T T T T T T T T T T T T T T    
1033	. . . . . . . . . . . . . . . . . C C C C C C C C C C C C C C C C C C C C C C C C    
1034	. . . . . . . . . . . . . . . . . C C C C C C C C C C C C C C C C C C C C C C C C    
1035	. . . . . . . . . . . . . . . . . A A A A A A A A A A A A A A A A A A A A A A A A    
1036	. . . . . . . . . . . . . . . . . T T T T T T T T T T T T T T T T T T T T T T T T    
1037	. . . . . . . . . . . . . . . . . T T T T T T T T T T T T T T T T T T T T T T T T    
1038	. . . . . . . . . . . . . . . . . T T T T T T T T T T T T T T T T T T T T T T T T    
1039	. . . . . . . . . . . . . . . . . G G G G G G G G G G G G G G G G G G G G G G G G    
1040	. . . . . . . . . . . . . . . . . T T T T T T T T T T T T T T T T T T T T T T T T    
1041	. . . . . . . . . . . . . . . . . T T T T T T T T T T T T T T T T T T T T T T T T    
1042	. . . . . . . . . . . . . . . . . T T T T T T T T T T T T T T T T T T T T T T T T    
1043	. . . . . . . . . . . . . . . . . G G G G G G G G G G G G G G G G G G G G G G G G    
1044	. . . . . . . . . . . . . . . . . T T T T T T T T T T T T T T T T T T T T T T T T    
1045	. . . . . . . . . . . . . . . . . C C C C C C C C C C C C C C C C C C C C C C C C    
1046	. . . . . . . . . . . . . . . . . T T T T T T T T T T T T T T T T T T T T T T T T    
1047	. . . . . . . . . . . . . . . . . G G G G G G G G G G G G G G G G G G G G G G G G    
1048	. . . . . . . . . . . . . . . . . T T T T T T T T T T T T T T T T T T T T T T T T    
1049	. . . . . . . . . . . . . . . . . - - - - - - - - - - - - C - - C - - - - - - - -    
1050	. . . . . . . . . . . . . . . . . - - - - - - - - - - - - T - - T - - - - - - - -    
1051	. . . . . . . . . . . . . . . . . - - - - - - - - - - - - G - - G - - - - - - - -    
1052	. . . . . . . . . . . . . . . . . - - - - - - - - - - - - T - - T - - - - - - - -    
1053	. . . . . . . . . . . . . . . . . A A A A A A A A A A A A A A A A A A A A A A A A    
1054	. . . . . . . . . . . . . . . . . T T T T T T T T T T T T T T T T T T T T T T T T    
1055	. . . . . . . . . . . . . . . . . T T T T T T T T T T T T T T T T T T T T T T T T    
1056	. . . . . . . . . . . . . . . . . T T T T T T T T T T T T T T T T T T T T T T T T    
1057	. . . . . . . . . . . . . . . . . T T T T T T T T T T T T T T T T T T T T T T T T    
1058	. . . . . . . . . . . . . . . . . A A A A A A A A A A A A A A A A A A A A A A A A    
1059	. . . . . . . . . . . . . . . . . T T T T T T T T T T T T T T T T T T T T T T T T    
1060	. . . . . . . . . . . . . . . . . G G G G G G G G G G G G G G G G G G G G G G G G    
1061	. . . . . . . . . . . . . . . . . T T T T T T T T T T T T T T T T T T T T T T T T    
1062	. . . . . . . . . . . . . . . . . T T T T T T T T T T T T T T T T T T T T T T T T    
1063	. . . . . . . . . . . . . . . . . A A A A A A A A A A A A A A A A A A A A A A A A    
1064	. . . . . . . . . . . . . . . . . A A A A A A A A A A A A A A A A A A A A A A A A    
1065	. . . . . . . . . . . . . . . . . G G G G G G G G G G G G G G G G G G G G G G G G    
1066	. . . . . . . . . . . . . . . . . T T T T T T T T T T T T T T T T T T T T T T T T    
1067	. . . . . . . . . . . . . . . . . A A A A A A A A A A A A A A A A A A A A A A A A    
1068	. . . . . . . . . . . . . . . . . A A A A A A A A A A A A A A A A A A A A A A A A    
1069	. . . . . . . . . . . . . . . . . C C C C C C C C C C C C C C C C C C C C C C C C    
1070	. . . . . . . . . . . . . . . . . A A A A A A A A A A A A A A A A A A A A A A A A    
1071	. . . . . . . . . . . . . . . . . C C C C C C C C C C C C C C C C C C C C C C C C    
1072	. . . . . . . . . . . . . . . . . T T T T T T T T T T T T T T T T T T T T T T T T    
1073	. . . . . . . . . . . . . . . . . T T T T T T T T T T T T T T T T T T T T T T T T    
1074	. . . . . . . . . . . . . . . . . G G G G G G G G G G G G G G G G G G G G G G G G    
1075	. . . . . . . . . . . . . . . . . T T T T T T T T T T T T T T T T T T T T T T T T    
1076	. . . . . . . . . . . . . . . . . G G G G G G G G G G G G G G G G G G G G G G G G    
1077	. . . . . . . . . . . . . . . . . A A A A A A A A A A A A A A A A A A A A A A A A    
1078	. . . . . . . . . . . . . . . . . C C C C - C C C C T C C C C C C C C C C C C C C    
1079	. . . . . . . . . . . . . . . . . A A A A - A A A A A A A A A A A A A A A A A A A    
1080	. . . . . . . . . . . . . . . . . C C C C C C C C C C C C C C C C C C C C C C C C    
1081	. . . . . . . . . . . . . . . . . A A A A A A A A A A A A A A A A A A A A A A A A    
1082	. . . . . . . . . . . . . . . . . C C C C C C C C C C C C C C C C C C C C C C C C    
1083	. . . . . . . . . . . . . . . . . A A A A A A A A A A A A A A A A A A A A A A A A    
1084	. . . . . . . . . . . . . . . . . C C C C C C C C C C C C C C C C C C C C C C C C    
1085	. . . . . . . . . . . . . . . . . A A A A A A A A A A A A A A A A A A A A A A A A    
1086	. . . . . . . . . . . . . . . . . C C C C C C C C C C C C C C C C C C C C C C C C    
1087	. . . . . . . . . . . . . . . . . A A A A A A A A A A A A A A A A A A A A A A A A    
1088	. . . . . . . . . . . . . . . . . C C C C C C C C C C C C C C C C C C C C C C C C    
1089	. . . . . . . . . . . . . . . . . A A T T T T T T T T T T T T T T T T T T T T T T    
1090	. . . . . . . . . . . . . . . . . . . C C C C C C C C C C C C C C C C C C C C C C    
1091	. . . . . . . . . . . . . . . . . . . A A A A A A A A A A A A A A A A A A A A A A    
1092	. . . . . . . . . . . . . . . . . . . G G G G G G G G G G G G G G G G G G G G G G    
1093	. . . . . . . . . . . . . . . . . . . A A A A A A A A A A A A A A A A A A A A A A    
1094	. . . . . . . . . . . . . . . . . . . T T T T T T T T T T T T T T T T T T T T T T    
1095	. . . . . . . . . . . . . . . . . . . A A A A A A A A A A A A A A A A A A A A A A    
1096	. . . . . . . . . . . . . . . . . . . C C C C C C C C C C C C C C C C C C C C C C    
1097	. . . . . . . . . . . . . . . . . . . C C C C C C C C C C C C C C C C C C C C C C    
1098	. . . . . . . . . . . . . . . . . . . A A A A A A A A A A A A A A A A A A A A A A    
1099	. . . . . . . . . . . . . . . . . . . G G G G G G G G G G G G G G G G G G G G G G    
1100	. . . . . . . . . . . . . . . . . . . T T T T T T T T T T T T T T T T T T T T T T    
1101	. . . . . . . . . . . . . . . . . . . G G G G G G G G G G G G G G G G G G G G G G    
1102	. . . . . . . . . . . . . . . . . . . A A A A A A A A A A A A A A A A A A A A A A    
1103	. . . . . . . . . . . . . . . . . . . A A A A A A A A A A A A A A A A A A A A A A    
1104	. . . . . . . . . . . . . . . . . . . T T T T T T T T T T T T T T T T T T T T T T    
1105	. . . . . . . . . . . . . . . . . . . G G G G G G G G G G G G G G G G G G G G G G    
1106	. . . . . . . . . . . . . . . . . . . G T T T T T T T T T T T T T T T T T T T T T    
1107	. . . . . . . . . . . . . . . . . . . . T T T T T T T T T T T T T T T T T T T T T    
1108	. . . . . . . . . . . . . . . . . . . . G G G G G G G G G G G G G G G G G G G G G    
1109	. . . . . . . . . . . . . . . . . . . . T T T T T T T T T T T T T T T T T T T T T    
1110	. . . . . . . . . . . . . . . . . . . . T T T T T T T T T T T T T T T T T T T T T    
1111	. . . . . . . . . . . . . . . . . . . . C C C C C C C C C C C C C C C C C C C C C    
1112	. . . . . . . . . . . . . . . . . . . . T T T T T T T T T T T T T T T T T T T T T    
1113	. . . . . . . . . . . . . . . . . . . . C C C C C C C C C C C C C C C C C C C C C    
1114	. . . . . . . . . . . . . . . . . . . . A A A A A A A A A A A A A A A A A A A A A    
1115	. . . . . . . . . . . . . . . . . . . . T T T T T T T T T T T T T T T T T T T T T    
1116	. . . . . . . . . . . . . . . . . . . . C C C C C C C C C C C C C C C C C C C C C    
1117	. . . . . . . . . . . . . . . . . . . . A A A A A A A A A A A A A A A A A A A A A    
1118	. . . . . . . . . . . . . . . . . . . . A A A A A A A A A A A A A A A A A A A A A    
1119	. . . . . . . . . . . . . . . . . . . . A A A A A A A A A A A A A A A A A A A A A    
1120	. . . . . . . . . . . . . . . . . . . . C C C C C C C C C C C C C C C C C C C C C    
1121	. . . . . . . . . . . . . . . . . . . . - - C C C C - - - - - - - C C C C C C C C  9	 indel  
1122	. . . . . . . . . . . . . . . . . . . . - - A A A A - - - - - - - A A A A A A A A  9	 indel  
1123	. . . . . . . . . . . . . . . . . . . . G G G G G G G G G G G G G G G G G G G G G    
1124	. . . . . . . . . . . . . . . . . . . . T T T T T T T T T T T T T T T T T T T T T    
1125	. . . . . . . . . . . . . . . . . . . . T T T T T T T T T T T T T T T T T T T T T    
1126	. . . . . . . . . . . . . . . . . . . . T T T T T T T T T T T T T T T T T T T T T    
1127	. . . . . . . . . . . . . . . . . . . . G G G G G G G G G G G G G G G G G G G G G    
1128	. . . . . . . . . . . . . . . . . . . . T T T T T T T T T T T T T T T T T T T T T    
1129	. . . . . . . . . . . . . . . . . . . . T T T T T T T T T T T T T T T T T T T T T    
1130	. . . . . . . . . . . . . . . . . . . . T T T T T T T T T T T T T T T T T T T T T    
1131	. . . . . . . . . . . . . . . . . . . . T T T T T T T T T T T T T T T T T T T T T    
1132	. . . . . . . . . . . . . . . . . . . . C C C C C C C C C C C C C C C C C C C C C    
1133	. . . . . . . . . . . . . . . . . . . . A A A A A A A A A A A A A A A A A A A A A    
1134	. . . . . . . . . . . . . . . . . . . . A A A A A A A A A A A A A A A A A A A A A    
1135	. . . . . . . . . . . . . . . . . . . . A A A A A A A A A A A A A A A A A A A A A    
1136	. . . . . . . . . . . . . . . . . . . . G G G G G G G G G G G G G G G G G G G G G    
1137	. . . . . . . . . . . . . . . . . . . . G G G G G G G G G G G G G G G G G G G G G    
1138	. . . . . . . . . . . . . . . . . . . . T T T T T T T T T T T T T T T T T T T T T    
1139	. . . . . . . . . . . . . . . . . . . . A A A A A A A A A A A A A A A A A A A A A    
1140	. . . . . . . . . . . . . . . . . . . . G G G G G G G G G G G G G G G G G G G G G    
1141	. . . . . . . . . . . . . . . . . . . . C C C C C C C C C C C C C C C C C C C C C    
1142	. . . . . . . . . . . . . . . . . . . . A A A A A A A A A A A A A A A A A A A A A    
1143	. . . . . . . . . . . . . . . . . . . . A A A A A A A A A A A A A A A A A A A A A    
1144	. . . . . . . . . . . . . . . . . . . . A A A A A A A A A A A A A A A A A A A A A    
1145	. . . . . . . . . . . . . . . . . . . . T T T T T T T T T T T T T T T T T T T T T    
1146	. . . . . . . . . . . . . . . . . . . . G G G G G G G G G G G G G G G G G G G G G    
1147	. . . . . . . . . . . . . . . . . . . . C C C C C C C C C C C C C C C C C C C C C    
1148	. . . . . . . . . . . . . . . . . . . . T T T T T T T T T T T T T T T T T T T T T    
1149	. . . . . . . . . . . . . . . . . . . . A A A A A A A A A A A A A A A A A A A A A    
1150	. . . . . . . . . . . . . . . . . . . . G G G G G G G G G G G G G G G G G G G G G    
1151	. . . . . . . . . . . . . . . . . . . . A A A A A A A A A A A A A A A A A A A A A    
1152	. . . . . . . . . . . . . . . . . . . . A A A A A A A A A A A A A A A A A A A A A    
1153	. . . . . . . . . . . . . . . . . . . . G G G G G G G G G G G G G G G G G G G G G    
1154	. . . . . . . . . . . . . . . . . . . . T T T T T T T T T T T T T T T T T T T T T    
1155	. . . . . . . . . . . . . . . . . . . . G G G G G G G G G G G G G G G G G G G G G    
1156	. . . . . . . . . . . . . . . . . . . . T T T T T T T T T T T T T T T T T T T T T    
1157	. . . . . . . . . . . . . . . . . . . . C C C C C C C C C C C C C C C C C C C C C    
1158	. . . . . . . . . . . . . . . . . . . . T T T T T T T T T T T T T T T T T T T T T    
1159	. . . . . . . . . . . . . . . . . . . . C C C C C C C C C C C C C C C C C C C C C    
1160	. . . . . . . . . . . . . . . . . . . . A A A A A A A A A A A A A A A A A A A A A    
1161	. . . . . . . . . . . . . . . . . . . . A A A A A A A A A A A A A A A A A A A A A    
1162	. . . . . . . . . . . . . . . . . . . . A A A A A A A A A A A A A A A A A A A A A    
1163	. . . . . . . . . . . . . . . . . . . . G G G G G G G G G G G G G G G G G G G G G    
1164	. . . . . . . . . . . . . . . . . . . . G G G G G G G G G G G G G G G G G G G G G    
1165	. . . . . . . . . . . . . . . . . . . . C C C C C C C C C C C C C C C C C C C C C    
1166	. . . . . . . . . . . . . . . . . . . . C C C C C C C C C C C C C C C C C C C C C    
1167	. . . . . . . . . . . . . . . . . . . . T T T T T T T T T T T T T T T T T T T T T    
1168	. . . . . . . . . . . . . . . . . . . . C C C C C C C C C C C C C C C C C C C C C    
1169	. . . . . . . . . . . . . . . . . . . . C C C C C C C C C C C C C C C C C C C C C    
1170	. . . . . . . . . . . . . . . . . . . . T T T T T T T T T T T T T T T T T T T T T    
1171	. . . . . . . . . . . . . . . . . . . . G G G G G G G G G G G G G G G G G G G G G    
1172	. . . . . . . . . . . . . . . . . . . . T T T T T T T T T T T T T T T T T T T T T    
1173	. . . . . . . . . . . . . . . . . . . . C C C C C C C C C C C C C C C C C C C C C    
1174	. . . . . . . . . . . . . . . . . . . . T T T T T T T T T T T T T T T T T T T T T    
1175	. . . . . . . . . . . . . . . . . . . . A A A A A A A A A A A A A A A A A A A A A    
1176	. . . . . . . . . . . . . . . . . . . . T T T T T T T T T T T T T T T T T T T T T    
1177	. . . . . . . . . . . . . . . . . . . . A A A A A A A A A A A A A A A A A A A A A    
1178	. . . . . . . . . . . . . . . . . . . . C C C C C C C C C C C C C C C C C C C C C    
1179	. . . . . . . . . . . . . . . . . . . . C C C C C C C C C C C C C C C C C C C C C    
1180	. . . . . . . . . . . . . . . . . . . . A A A A A A A A A A A A A A A A A A A A A    
1181	. . . . . . . . . . . . . . . . . . . . T T T T T T T T T T T T T T T T T T T T T    
1182	. . . . . . . . . . . . . . . . . . . . A A A A A A A A A A A A A A A A A A A A A    
1183	. . . . . . . . . . . . . . . . . . . . A A A A A A A A A A A A A A A A A A A A A    
1184	. . . . . . . . . . . . . . . . . . . . A A A A A A A A A A A A A A A A A A A A A    
1185	. . . . . . . . . . . . . . . . . . . . A A A A A A A A A A A A A A A A A A A A A    
1186	. . . . . . . . . . . . . . . . . . . . T T T T T T T T T T T T T T T T T T T T T    
1187	. . . . . . . . . . . . . . . . . . . . . C C C C C C C C C C C C C C C C C C C C    
1188	. . . . . . . . . . . . . . . . . . . . . A A A A A A A A A A A A A A A A A A A A    
1189	. . . . . . . . . . . . . . . . . . . . . C C C C C C C C C C C C C C C C C C C C    
1190	. . . . . . . . . . . . . . . . . . . . . A A A A A A A A A A A A A A A A A A A A    
1191	. . . . . . . . . . . . . . . . . . . . . C C C C C C C C C C C C C C C C C C C C    
1192	. . . . . . . . . . . . . . . . . . . . . A A A A A A A A A A A A A A A A A A A A    
1193	. . . . . . . . . . . . . . . . . . . . . C C C C C C C C C C C C C C C C C C C C    
1194	. . . . . . . . . . . . . . . . . . . . . A A A A A A A A A A A A A A A A A A A A    
1195	. . . . . . . . . . . . . . . . . . . . . C C C C C C C C C C C C C C C C C C C C    
1196	. . . . . . . . . . . . . . . . . . . . . A A A A A A A A A A A A A A A A A A A A    
1197	. . . . . . . . . . . . . . . . . . . . . C C C C C C C C C C C C C C C C C C C C    
1198	. . . . . . . . . . . . . . . . . . . . . A A A A A A A A A A A A A A A A A A A A    
1199	. . . . . . . . . . . . . . . . . . . . . A A A A A A A A A A A A A A A A A A A A    
1200	. . . . . . . . . . . . . . . . . . . . . A A A A A A A A A A A A A A A A A A A A    
1201	. . . . . . . . . . . . . . . . . . . . . C C C C C C C C C C C C C C C C C C C C    
1202	. . . . . . . . . . . . . . . . . . . . . A A A A A A A A A A A A A A A A A A A A    
1203	. . . . . . . . . . . . . . . . . . . . . G G G G G G G G G G A G G G G G G G G G    
1204	. . . . . . . . . . . . . . . . . . . . . G G G G G G G G G G G G G G G G G G G G    
1205	. . . . . . . . . . . . . . . . . . . . . A A A A A A A A A A A A A A A A A A A A    
1206	. . . . . . . . . . . . . . . . . . . . . A A A A A A A A A A A A A A A A A A A A    
1207	. . . . . . . . . . . . . . . . . . . . . G G G G G G G G G G G G G G G G G G G G    
1208	. . . . . . . . . . . . . . . . . . . . . A A A A A A A A A A A A A A A A A A A A    
1209	. . . . . . . . . . . . . . . . . . . . . A A A A A A A A A A A A A A A A A A A A    
1210	. . . . . . . . . . . . . . . . . . . . . G G G G G G G G G G G G G G G G G G G G    
1211	. . . . . . . . . . . . . . . . . . . . . G G G G G G G G G G G G G G G G G G G G    
1212	. . . . . . . . . . . . . . . . . . . . . A A A A A A A A A A A A A A A A A A A A    
1213	. . . . . . . . . . . . . . . . . . . . . A A A A A A A A A A A A A A A A A A A A    
1214	. . . . . . . . . . . . . . . . . . . . . A A A A A A A A A A A A A A A A A A A A    
1215	. . . . . . . . . . . . . . . . . . . . . C C C C C C C C C C C C C C C C C C C C    
1216	. . . . . . . . . . . . . . . . . . . . . C C C C C C C C C C C C C C C C C C C C    
1217	. . . . . . . . . . . . . . . . . . . . . G G G G G G G G G G G G G G G G G G G G    
1218	. . . . . . . . . . . . . . . . . . . . . G T T T T T T T T T T T T T T T T T T T    
1219	. . . . . . . . . . . . . . . . . . . . . . G G G G G G G G G G G G G G G G G G G    
1220	. . . . . . . . . . . . . . . . . . . . . . T T T T T T T T T T T T T T T T T T T    
1221	. . . . . . . . . . . . . . . . . . . . . . A A A A A A A A A A A A A A A A A A A    
1222	. . . . . . . . . . . . . . . . . . . . . . C C C C C C C C C C C C C C C C C C C    
1223	. . . . . . . . . . . . . . . . . . . . . . A A A A A A A A A A A A A A A A A A A    
1224	. . . . . . . . . . . . . . . . . . . . . . A A A A A A A A A A A A A A A A A A A    
1225	. . . . . . . . . . . . . . . . . . . . . . A A A A A A A A A A A A A A A A A A A    
1226	. . . . . . . . . . . . . . . . . . . . . . A A A A A A A A A A A A A A A A A A A    
1227	. . . . . . . . . . . . . . . . . . . . . . T T T T T T T T T T T T T T T T T T T    
1228	. . . . . . . . . . . . . . . . . . . . . . C C C C C C C C C C C C C C C C C C C    
1229	. . . . . . . . . . . . . . . . . . . . . . A A A A A A A A A A A A A A A A A A A    
1230	. . . . . . . . . . . . . . . . . . . . . . C C C C C C C C C C C C C C C C C C C    
1231	. . . . . . . . . . . . . . . . . . . . . . T T T T T T T T T T T T T T T T T T T    
1232	. . . . . . . . . . . . . . . . . . . . . . C C C C C C C C C C C C C C C C C C C    
1233	. . . . . . . . . . . . . . . . . . . . . . A A A A A A A A A A A A A A A A A A A    
1234	. . . . . . . . . . . . . . . . . . . . . . C C C C C C C C C C C C C C C C C C C    
1235	. . . . . . . . . . . . . . . . . . . . . . A A A A A A A A A A A A A A A A A A A    
1236	. . . . . . . . . . . . . . . . . . . . . . T T T T T T T T T T T T T T T T T T T    
1237	. . . . . . . . . . . . . . . . . . . . . . T T T T T T T T T T T T T T T T T T T    
1238	. . . . . . . . . . . . . . . . . . . . . . T T T T T T T T T T T T T T T T T T T    
1239	. . . . . . . . . . . . . . . . . . . . . . T T T T T T T T T T T T T T T T T T T    
1240	. . . . . . . . . . . . . . . . . . . . . . C C C C C C C C C C C C C C C C C C C    
1241	. . . . . . . . . . . . . . . . . . . . . . A A A A A A A A A A A A A A A A A A A    
1242	. . . . . . . . . . . . . . . . . . . . . . C C C C C C C C C C C C C C C C C C C    
1243	. . . . . . . . . . . . . . . . . . . . . . T T T T T T T T T T T T T T T T T T T    
1244	. . . . . . . . . . . . . . . . . . . . . . C C C C C C C C C C C C C C C C C C C    
1245	. . . . . . . . . . . . . . . . . . . . . . G G G G G G G G G G G G G G G G G G G    
1246	. . . . . . . . . . . . . . . . . . . . . . C C C C C C C C C C C C C C C C C C C    
1247	. . . . . . . . . . . . . . . . . . . . . . A A A A A A A A A A A A A A A A A A A    
1248	. . . . . . . . . . . . . . . . . . . . . . C C C C C C C C C C C C C C C C C C C    
1249	. . . . . . . . . . . . . . . . . . . . . . T T T T T T T T T T T T T T T T T T T    
1250	. . . . . . . . . . . . . . . . . . . . . . T T T T T T T T T T T T T T T T T T T    
1251	. . . . . . . . . . . . . . . . . . . . . . T T T T T T T T T T T T T T T T T T T    
1252	. . . . . . . . . . . . . . . . . . . . . . G G G G G G G G G G G G G G G G G G G    
1253	. . . . . . . . . . . . . . . . . . . . . . A A A A A A A A A A A A A A A A A G A    
1254	. . . . . . . . . . . . . . . . . . . . . . C C C C C C C C C C C C C C C C C C C    
1255	. . . . . . . . . . . . . . . . . . . . . . A A A A A A A A A A A A A A A A A A A    
1256	. . . . . . . . . . . . . . . . . . . . . . T T T T T T T T T T T T T T T T T T T    
1257	. . . . . . . . . . . . . . . . . . . . . . T T T T T T T T T T T T T T T T T T T    
1258	. . . . . . . . . . . . . . . . . . . . . . T T T T T T T T T T T T T T T T T T T    
1259	. . . . . . . . . . . . . . . . . . . . . . G G G G G G G G G G G G G G G G G G G    
1260	. . . . . . . . . . . . . . . . . . . . . . T T T T T T T T T T T T T T T T T T T    
1261	. . . . . . . . . . . . . . . . . . . . . . G G G G G G G G G G G G G G G G G G G    
1262	. . . . . . . . . . . . . . . . . . . . . . A A A A A A A A A A A A A A A A A A A    
1263	. . . . . . . . . . . . . . . . . . . . . . G G G G G G G G G G G G G G G G G G G    
1264	. . . . . . . . . . . . . . . . . . . . . . T T T T T T T T T T T T T T T T T T T    
1265	. . . . . . . . . . . . . . . . . . . . . . A A A A A A A A A A A A A A A A A A A    
1266	. . . . . . . . . . . . . . . . . . . . . . T T T T T T T T T T T T C T T T T T T    
1267	. . . . . . . . . . . . . . . . . . . . . . G G G G G G G G G G G G G G G G G G G    
1268	. . . . . . . . . . . . . . . . . . . . . . C C C C C C C C C C C C C C C C C C C    
1269	. . . . . . . . . . . . . . . . . . . . . . A A A A A A A A A A A A A A A A A A A    
1270	. . . . . . . . . . . . . . . . . . . . . . C C C C C C C C C C C C C C C C C C C    
1271	. . . . . . . . . . . . . . . . . . . . . . A A A A A A A A A A A A A A A A A A A    
1272	. . . . . . . . . . . . . . . . . . . . . . T T T T T T T T T T T T T T T T T T T    
1273	. . . . . . . . . . . . . . . . . . . . . . T T T T T T T T T T T T T T T T T T T    
1274	. . . . . . . . . . . . . . . . . . . . . . A A A A A A A A A A A A A A A A A A A    
1275	. . . . . . . . . . . . . . . . . . . . . . A A A A A A A A A A A A A A A A A A A    
1276	. . . . . . . . . . . . . . . . . . . . . . T T T T T T T T T T T T T T T T T T T    
1277	. . . . . . . . . . . . . . . . . . . . . . G G G G G G G G G G G G G G G G G G G    
1278	. . . . . . . . . . . . . . . . . . . . . . A A A A A A A A A A A A A A A A A A A    
1279	. . . . . . . . . . . . . . . . . . . . . . A A A A A A A A A A A A A A A A A A A    
1280	. . . . . . . . . . . . . . . . . . . . . . T T T T T T T T T T T T T T T T T T T    
1281	. . . . . . . . . . . . . . . . . . . . . . T T T T T T T T T T T T T T T T T T T    
1282	. . . . . . . . . . . . . . . . . . . . . . C C C C C C C C C C C C C C C C C C C    
1283	. . . . . . . . . . . . . . . . . . . . . . A A A A A A A A A A A A A A A A A A A    
1284	. . . . . . . . . . . . . . . . . . . . . . T T T T T T T T T T T T T T T T T T T    
1285	. . . . . . . . . . . . . . . . . . . . . . G G G G G G G G G A G G G G G G G G G    
1286	. . . . . . . . . . . . . . . . . . . . . . T T T T T T T T T T T T T T T T T T T    
1287	. . . . . . . . . . . . . . . . . . . . . . A A A A A A A A A A A A A A A A A A A    
1288	. . . . . . . . . . . . . . . . . . . . . . C C C C C C C C C C C C C C C C C C C    
1289	. . . . . . . . . . . . . . . . . . . . . . G G G G G G G G G G G G G G G G G G G    
1290	. . . . . . . . . . . . . . . . . . . . . . T T T T T T T T T T T T T T T T T T T    
1291	. . . . . . . . . . . . . . . . . . . . . . G G G G G G G G G G G G G G G G G G G    
1292	. . . . . . . . . . . . . . . . . . . . . . A A A A A A A A A A A A A A A A A A A    
1293	. . . . . . . . . . . . . . . . . . . . . . A A A A A A A A A A A A A A A A A A A    
1294	. . . . . . . . . . . . . . . . . . . . . . C C C C C C C C C C C C C C C C C C C    
1295	. . . . . . . . . . . . . . . . . . . . . . A A A A A A A A A A A A A A A A A A A    
1296	. . . . . . . . . . . . . . . . . . . . . . G G G G G G G G G G G G G G G G G G G    
1297	. . . . . . . . . . . . . . . . . . . . . . A A A A A A A A A A A A A A A A A A A    
1298	. . . . . . . . . . . . . . . . . . . . . . A A A A A A A A A A A A A A A A A A A    
1299	. . . . . . . . . . . . . . . . . . . . . . A A A A A A A A A A A A A A A A A A A    
1300	. . . . . . . . . . . . . . . . . . . . . . A A A A A A A A A A A A A A A A A A A    
1301	. . . . . . . . . . . . . . . . . . . . . . T T T T T T T T T T T T T T T T T T T    
1302	. . . . . . . . . . . . . . . . . . . . . . A A A A A A A A A A A A A A A A A A A    
1303	. . . . . . . . . . . . . . . . . . . . . . C C C C C C C C C C C C C C C C C C C    
1304	. . . . . . . . . . . . . . . . . . . . . . A A A A A A A A A A A A A A A A A A A    
1305	. . . . . . . . . . . . . . . . . . . . . . A A A A A A A A A A A A A A A A A A A    
1306	. . . . . . . . . . . . . . . . . . . . . . T T T T T T T T T T T T T T T T T T T    
1307	. . . . . . . . . . . . . . . . . . . . . . T T T T T T T T T T T T T T T T T T T    
1308	. . . . . . . . . . . . . . . . . . . . . . T T T T T T T T T T T T T T T T T T T    
1309	. . . . . . . . . . . . . . . . . . . . . . C C A C C C C C C C C C C C C C C C C    
1310	. . . . . . . . . . . . . . . . . . . . . . C C . C C C C C C C C C C C C C C C C    
1311	. . . . . . . . . . . . . . . . . . . . . . C C . C T T T T T T T T T T C C C T T  6	 transition  
1312	. . . . . . . . . . . . . . . . . . . . . . C . . C C C C C C C C C C C C C C C C    
1313	. . . . . . . . . . . . . . . . . . . . . . T . . T T T T T T T T T T T T T T T T    
1314	. . . . . . . . . . . . . . . . . . . . . . T . . . T T T T T T T T T T T T T T T    
1315	. . . . . . . . . . . . . . . . . . . . . . T . . . T T T T T T T T T T T T T T T    
1316	. . . . . . . . . . . . . . . . . . . . . . T . . . T T T T T T T T T T T T T T T    
1317	. . . . . . . . . . . . . . . . . . . . . . T . . . T T T T T T T T T T T T T T T    
1318	. . . . . . . . . . . . . . . . . . . . . . . . . . C C C C C C C C C C C C C C C    
1319	. . . . . . . . . . . . . . . . . . . . . . . . . . T T T T T T T T T T T T T T T    
1320	. . . . . . . . . . . . . . . . . . . . . . . . . . T T T T T T T T T T T T T T T    
1321	. . . . . . . . . . . . . . . . . . . . . . . . . . T T T T T T T T T T T T T T T    
1322	. . . . . . . . . . . . . . . . . . . . . . . . . . G G G G G G G G G G G G G G G    
1323	. . . . . . . . . . . . . . . . . . . . . . . . . . T T T T T T T T T T T T T T T    
1324	. . . . . . . . . . . . . . . . . . . . . . . . . . T T T T T T T T T T T T T T T    
1325	. . . . . . . . . . . . . . . . . . . . . . . . . . T T T T T T T T T T T T T T T    
1326	. . . . . . . . . . . . . . . . . . . . . . . . . . T T T T T T T T T T T T T T T    
1327	. . . . . . . . . . . . . . . . . . . . . . . . . . G G G G G G G G G G G G G G G    
1328	. . . . . . . . . . . . . . . . . . . . . . . . . . T T T T T T T T T T T T T T T    
1329	. . . . . . . . . . . . . . . . . . . . . . . . . . T T T T T T T T T T T T T T T    
1330	. . . . . . . . . . . . . . . . . . . . . . . . . . G G G G G G G G G G G G G G G    
1331	. . . . . . . . . . . . . . . . . . . . . . . . . . T T T T T T T T T T T T T T T    
1332	. . . . . . . . . . . . . . . . . . . . . . . . . . T T T T T T T T T T T T T T T    
1333	. . . . . . . . . . . . . . . . . . . . . . . . . . C C C C C C C C C C C C C C C    
1334	. . . . . . . . . . . . . . . . . . . . . . . . . . C C C C C C C C C C C C C C C    
1335	. . . . . . . . . . . . . . . . . . . . . . . . . . A A A A A A A A A A A A A A A    
1336	. . . . . . . . . . . . . . . . . . . . . . . . . . T T T T T T T T T T T T T T T    
1337	. . . . . . . . . . . . . . . . . . . . . . . . . . G G G G G G G G G G G G G G G    
1338	. . . . . . . . . . . . . . . . . . . . . . . . . . T T T T T T T T T T T T T T T    
1339	. . . . . . . . . . . . . . . . . . . . . . . . . . T T T T T T T T T T T T T T T    
1340	. . . . . . . . . . . . . . . . . . . . . . . . . . T T T T T T T T T T T T T T T    
1341	. . . . . . . . . . . . . . . . . . . . . . . . . . C C C C C C C C C C C C C C C    
1342	. . . . . . . . . . . . . . . . . . . . . . . . . . T T T T T T T T T T T T T T T    
1343	. . . . . . . . . . . . . . . . . . . . . . . . . . G G G G G G G G G G G G G G G    
1344	. . . . . . . . . . . . . . . . . . . . . . . . . . T T T T T T T T T T T T T T T    
1345	. . . . . . . . . . . . . . . . . . . . . . . . . . A A A A A A A A A A A A A A A    
1346	. . . . . . . . . . . . . . . . . . . . . . . . . . A A A A A A A A A A A A A A A    
1347	. . . . . . . . . . . . . . . . . . . . . . . . . . G G G G G G G G G G G G G G G    
1348	. . . . . . . . . . . . . . . . . . . . . . . . . . T T T T T T T T T T T T T T T    
1349	. . . . . . . . . . . . . . . . . . . . . . . . . . A A A A A A A A A A A A A A A    
1350	. . . . . . . . . . . . . . . . . . . . . . . . . . A A A A A A A A A A A A A A A    
1351	. . . . . . . . . . . . . . . . . . . . . . . . . . T T T T T T T T T T T T T T T    
1352	. . . . . . . . . . . . . . . . . . . . . . . . . . A A A A A A A A A A A A A A A    
1353	. . . . . . . . . . . . . . . . . . . . . . . . . . A A A A A A A A A A A A A A A    
1354	. . . . . . . . . . . . . . . . . . . . . . . . . . A A A A A A A A A A A A A A A    
1355	. . . . . . . . . . . . . . . . . . . . . . . . . . T T T T T T T T T T T T T T T    
1356	. . . . . . . . . . . . . . . . . . . . . . . . . . A A A A A A A A A A A A A A A    
1357	. . . . . . . . . . . . . . . . . . . . . . . . . . A A A A A A A A A A A A A A A    
1358	. . . . . . . . . . . . . . . . . . . . . . . . . . T T T T T T T T T T T T T T T    
1359	. . . . . . . . . . . . . . . . . . . . . . . . . . C C C C C C C C C C C C C C C    
1360	. . . . . . . . . . . . . . . . . . . . . . . . . . T T T T T T T T T T T T T T T    
1361	. . . . . . . . . . . . . . . . . . . . . . . . . . G G G G G G G G G G G G G G G    
1362	. . . . . . . . . . . . . . . . . . . . . . . . . . C C C C C C C C C C C C C C C    
1363	. . . . . . . . . . . . . . . . . . . . . . . . . . T T T T T T T T T T T T T T T    
1364	. . . . . . . . . . . . . . . . . . . . . . . . . . G G G G G G G G G G G G G G G    
1365	. . . . . . . . . . . . . . . . . . . . . . . . . . A A A A A A G A A A A A A A A    
1366	. . . . . . . . . . . . . . . . . . . . . . . . . . T T T T T T . T T T T T T T T    
1367	. . . . . . . . . . . . . . . . . . . . . . . . . . T T T T T T . T T T T T T T T    
1368	. . . . . . . . . . . . . . . . . . . . . . . . . . T T T T T T . T T T T T T T T    
1369	. . . . . . . . . . . . . . . . . . . . . . . . . . T T T T T T . T T T T T . T T    
1370	. . . . . . . . . . . . . . . . . . . . . . . . . . T T T T T T . T T T T T . T T    
1371	. . . . . . . . . . . . . . . . . . . . . . . . . . . . T . . . . . . . . . . . .
```
